# Botulinum Toxin for Bruxism: An Overview

**DOI:** 10.3390/toxins17050249

**Published:** 2025-05-16

**Authors:** Manuella Salm Coelho, Júlia Meller Dias de Oliveira, Helena Polmann, Patrícia Pauletto, Cristine Miron Stefani, Lara Catarine De Luca Maciel, Graziela De Luca Canto

**Affiliations:** 1 Brazilian Centre for Evidence-Based Research (COBE), Department of Dentistry, Federal University of Santa Catarina (UFSC), Florianópolis 88040-535, Brazil; manuella.salm@gmail.com (M.S.C.); julia_meller5@hotmail.com (J.M.D.d.O.); hpolmann@hotmail.com (H.P.); delucacanto@gmail.com (G.D.L.C.); 2 Faculty of Dentistry, Universidad de las Americas (UDLA), Quito 170513, Ecuador; 3 Department of Dentistry, University of Brasília, Brasília 70910-900, Brazil; cmstefani@unb.br; 4 Centre for Cell Biology & Cutaneous Research, Blizard Institute, Queen Mary University of London, London E1 2AT, UK; laradlm@gmail.com; 5 Emergency Medicine and Evidence-Based Medicine, Federal University of São Paulo, São Paulo 040023-062, Brazil

**Keywords:** bruxism, botulinum toxins type A, masseter muscle, temporal muscle, overview, evidence-based dentistry

## Abstract

This overview aimed at assessing botulinum toxin type A (BoNT-A)’s effectiveness in managing bruxism compared to a placebo, the absence of treatment, or other interventions in adults. Only systematic reviews (SRs), with or without a meta-analysis, were included. A comprehensive literature search was conducted on 16 June 2024, encompassing seven databases and grey literature sources. Experts and reference lists of the included SRs were also consulted. Study selection was conducted in two phases by two independent authors. Methodological quality was evaluated using AMSTAR-2. Overlap was assessed using the corrected covered area. The 14 included SRs addressed several outcomes. In most studies, BoNT-A showed effectiveness in reducing pain (*n* = 10), the frequency of bruxism events (*n* = 7), and the maximum bite force (*n* = 5). None of the SRs effectively evaluated BoNT-A’s impact on functional movements. All of the included SRs scored “critically low” and “low” confidence rates in AMSTAR-2. Only one SR attempted to assess the certainty of the evidence, although unsuccessfully. The overlap across included SRs was rated as “high”, with a corrected covered area of 10.11%. The current systematic reviews on BoNT-A for bruxism lack methodological quality, limiting the reliability of their findings. Despite evidence indicating a potential reduction in pain, bruxism events, and the maximum bite force, methodological limitations prevent definitive conclusions from being drawn. High-quality research with standardized methodologies is essential to establish BoNT-A’s efficacy and support evidence-based clinical practice.

## 1. Introduction

Bruxism is a repetitive masticatory muscle activity that may involve teeth clenching or grinding and/or forceful mandibular movements [1]. It can be categorized according to the patient’s consciousness state as awake bruxism (AB) and sleep bruxism (SB) [1,2].

Recent literature suggests that bruxism is a pathophysiology that involves both central and peripheral mechanisms, where neurotransmitters modulate motor activity during different sleep stages [3]. Bruxism is often associated with transient arousals during light non-REM sleep, where excitatory neurotransmitters temporarily overcome inhibitory influences, leading to jaw muscle activation [3]. Additionally, genetic predisposition [4], stress, and psychosocial factors contribute to the susceptibility and manifestation of bruxism [5,6,7,8,9,10,11]. Specific factors include neurotransmitter activity (serotonin, dopamine, and norepinephrine), drug abuse, smoking, caffeine and alcohol intake, anxiety, and certain medications, like benzodiazepines, neuroleptics, amphetamines, tricyclic antidepressants, and selective serotonin reuptake inhibitors [5,6,7,8,9,10,11].

Prevalence rates of bruxism in adults vary from 8 to 31% for SB and 22 to 31% for AB [12,13,14,15]. This wide range may be attributed to discrepancies in studies’ methodologies, variations in the populations studied, and differences in diagnostic criteria [15]. Bruxism can result in several consequences, such as tooth wear, dental fractures, restoration failure, muscle hypertrophy, headaches, prominent linea alba, vertical enamel craze lines, and soft-tissue and periodontal alterations [11,16,17,18,19,20,21]. To prevent these issues, bruxism can be managed through different interventions, including oral splints, drug therapies (e.g., benzodiazepines, anticonvulsants, beta-blockers, serotonergic and dopaminergic agents, antidepressants, and muscle relaxants), biofeedback therapies, cannabidiol, transcutaneous electrical nerve stimulation, and other treatments (e.g., prosthetic rehabilitation and psychological therapies) [5,7,10,17,22,23].

Since the 1990s, the administration of BoNT-A has been one of the available pharmacological interventions for bruxism management [24]. BoNT-A functions by blocking acetylcholine release from presynaptic nerve endings in striated muscle, providing temporary muscular paralysis [25]. Previous literature has explored management approaches for bruxism, but none of the available overviews [5,16,17,22,26] specifically addressed the efficacy of BoNT-A. The existing systematic reviews (SRs) [23,27,28,29,30,31,32,33,34,35,36,37,38,39] have yielded controversial results and often misinterpreted bruxism by conflating it with conditions like temporomandibular dysfunction (TMD).

Given the ongoing uncertainty surrounding the efficacy of this intervention and the potential for its overuse in clinical practice, this overview was conducted to critically assess the SRs regarding the effectiveness of BoNT-A for managing bruxism in adults compared to a placebo, no treatment, or conventional therapies. Understanding its efficacy is essential to ensure evidence-based clinical decision-making and to prevent the inappropriate application of BoNT-A in bruxism management.

## 2. Results

### 2.1. Study Selection

The electronic database search initially identified 461 references. An additional 196 papers were sourced from grey literature (*n* = 180), and reference lists (*n* = 16). No additional paper was identified with expert consultation. After removing duplicates, 344 references remained.

**Figure 1:**
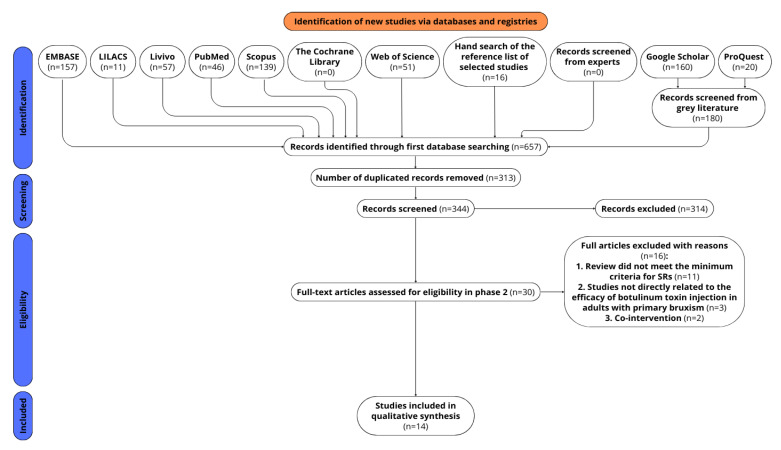
Flow diagram of literature search and selection criteria (adapted from Preferred Reporting Items for Systematic Reviews and Meta-Analysis). Figure generated with canva.com “URL: https://www.canva.com/design/DAGIcjM-HMg/WYI5w-0MDjjWZhTbRW6WyA/edit?utm_content=DAGIcjM-HMg&utm_campaign=designshare&utm_medium=link2&utm_source=sharebutton (accessed on 16 April 2025)”.

During phase 1, the screening of titles and abstracts, 314 studies were excluded. Subsequently, 30 registries underwent a full-text review (phase 2), leading to the inclusion of 14 SRs [23,27,28,29,30,31,32,33,34,35,36,37,38,39]. Figure 1 illustrates the process of study identification and selection.

Supplementary Material S1 shows the excluded primary studies and the reasons for exclusion.

### 2.2. Characteristics of Systematic Reviews

The SRs included in this study were published in 13 different journals between 2012 and 2023, and all studies were written in English. These reviews were carried out in several countries: Brazil [30,36,37,38], India [35], Italy [23], Republic of China [29,32,39], Romania [33], Spain [31], Sweden [27], United Kingdom [34], and Taiwan [28]. Detailed information regarding the characteristics of these SRs is available in Supplementary Material S2 and Supplementary Material S3.

### 2.3. Bibliometric Analysis

The summary of the bibliometric analysis can be found in Supplementary Material S4.

### 2.4. Methodological Quality

**Figure 2:**
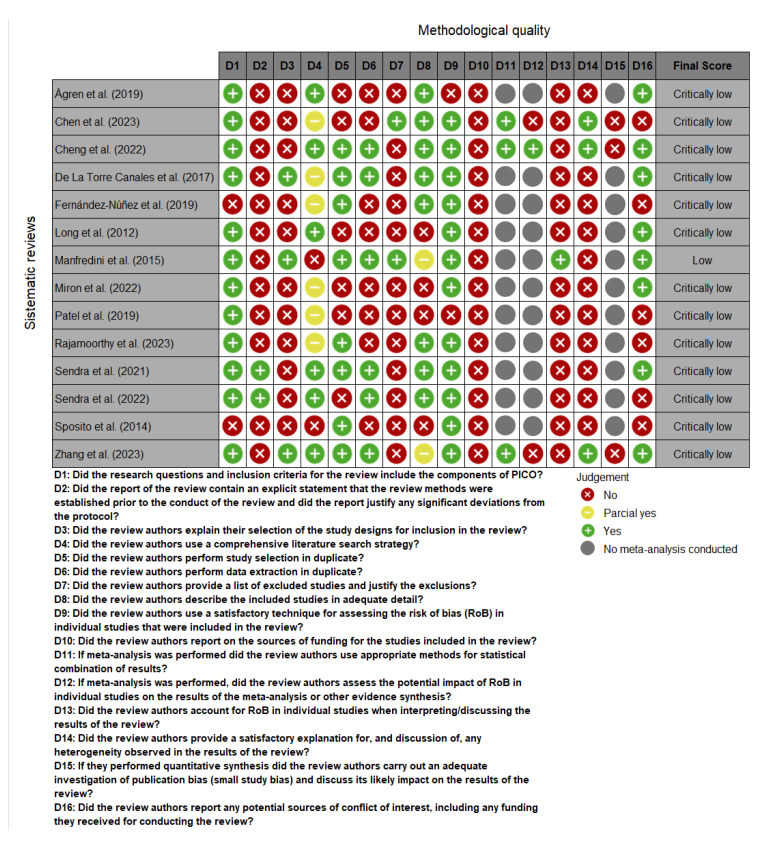
Methodological quality assessment using the AMSTAR-2 tool. Figure generated with Microsoft Excel [23,27,28,29,30,31,32,33,34,35,36,37,38,39].

All included SRs scored a “critically low” confidence rate, except for one that scored “low” [23]. The most critical domains were the statement of methods, comprehensive literature search strategy, justification of exclusions, risk of bias assessment, statistical combination of results, and interpretation of risk of bias in individual studies. Further details on the methodological quality assessment can be found in Figure 2.

### 2.5. Primary Study Overlap

**Figure 3:**
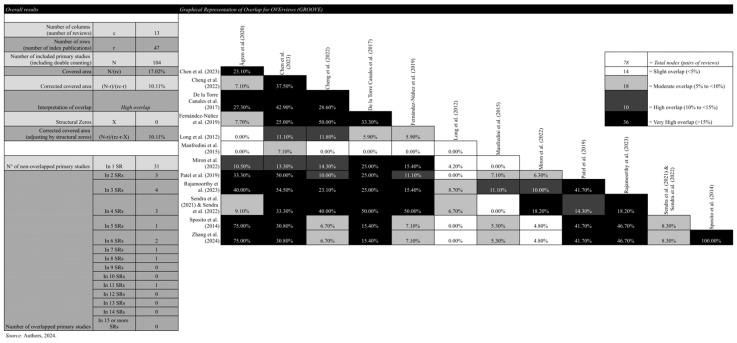
Graphical Representation of Overlap for OVErviews (GROOVE). Figure generated with Microsoft Excel [23,27,28,29,30,31,32,33,34,35,36,37,38,39].

In total, 48 primary studies were identified within the included SRs. The overall overlap across SRs was qualified as high with a corrected covered area of 10.11%. Figure 3 contains the GROOVE [40,41,42], while Supplementary Material S5 provides a detailed evidence matrix, listing the included studies within each systematic review.

### 2.6. Synthesis of Results of Systematic Reviews

Long et al. (2012) reviewed two RCTs and two controlled before-and-after studies, concluding that BoNT-A injections effectively reduced bruxism frequency and associated pain levels [32]. However, the authors emphasized the need for additional RCTs to further investigate the efficacy of BoNT-A in this context [32].

Sposito et al. (2014) found that BoNT-A injections on masseter and temporalis muscles effectively reduced pain levels and decreased bruxism episodes without significant adverse effects [38]. However, they highlighted the necessity for further research to confirm these findings [38].

Manfredini et al. (2015) reviewed two randomized controlled trials (RCTs) [23]. The authors noted that there is still insufficient evidence to recommend a standard treatment for sleep bruxism, aside from the use of oral appliances [23]. Notably, they did not conclude that botulinum toxin was effective for any of the outcomes assessed in this overview, including a reduction in the number of bruxism events, changes in pain, functional movement, or maximum bite force.

De la Torre Canales et al. (2017) included five studies: one took into account patients’ perspective after the intervention, two demonstrated a reduction in pain in the BoNT-A group, and two had mixed results on the number of bruxism events [30]. In relation to pain, the authors stated that the findings remain inconclusive [30]. Regarding the reduction of bruxism events, they noted that BoNT-A seemed to decrease the intensity of contractions associated with repetitive mandibular movements (RMMAs), rather than reducing the total number of episodes [30].

Fernández-Núñez et al. (2019) assessed four RCTs, which showed that BoNT-A is an effective intervention for reducing the frequency of bruxism episodes and decreasing pain levels and maximum occlusal force [31].

Patel et al. (2019) reviewed eleven trials and found that BoNT-A injections led to reductions in pain scores, the frequency of bruxism events, and the maximum bite force [34]. They noted variability in the effects on mouth opening without concluding whether BoNT-A was definitely effective for this outcome [34]. They emphasized that primary conservative treatments, such as self-management and patient education, should be exhausted before considering BoNT-A as an option [34].

Ågren et al. (2020) [27] reviewed four primary studies: three focused on the number of bruxism events and one focused on the maximum bite force. One study found no significant reduction in EMG activity after treatment, while the other two observed initial reductions in EMG activity, but no long-term changes [27]. The study measuring bite force reported significant decreases over time, with variations noted at different follow-up intervals (differences were notable at one week and one month, but not at six months) [27]. The authors stated that the efficacy of treatments in reducing bruxism events and maximum bite force remains inconclusive [27].

Sendra et al. (2021) and Sendra et al. (2022) reviewed multiple trials, affirming that BoNT-A effectively reduced myofascial pain symptoms in bruxism patients and was generally well-tolerated, though heterogeneous study designs precluded a meta-analysis [36,37].

Cheng et al. (2022) evaluated six articles. The BoNT-A group demonstrated an improvement in pain at chewing and pain at rest compared to the placebo group, calling for longer-term studies to assess recurrence risks [29]. Regarding bruxism events, the authors emphasized the necessity for further research to determine whether these events might recur or rebound after treatment [29]. Overall, they concluded that BoNT-A is an effective intervention for reducing both pain and the frequency of bruxism events [29].

Miron et al. (2022) reported that BoNT-A effectively decreased the frequency and duration of bruxism episodes, although it did not address the central mechanisms responsible for the condition [33]. The study also highlighted that localized muscle paralysis helped safeguard orofacial structures from excessive forces [33].

Chen et al. (2023) evaluated ten studies, demonstrating maximum bite force and pain reduction [28]. Despite heterogeneity among the studies, their statistical analysis showed that bite force reduction was effective for up to three months, while pain relief lasted up to 24 weeks [28]. Additionally, they found a dose-response relationship [28].

Rajamoorthy et al. (2023) evaluated six studies and highlighted that BoNT-A significantly decreased the bruxism frequency, associated pain levels, and maximum occlusal force compared to the placebo or traditional treatments [35].

Zhang et al. (2024) assessed data from eleven primary studies and performed a meta-analysis [39]. The meta-analysis revealed that the BoNT-A injections alleviated bruxism-related pain (with an average decrease of 4.06 points on the VAS immediately after treatment) [39]. The Bayesian analysis further confirmed these findings, indicating that BoNT-A was more effective than both oral splinting and saline injections [39].

### 2.7. Certainty Assessment

Of the 14 included SRs, only Sendra et al. (2022) [37] assessed the certainty of evidence using the GRADE approach [43]. The authors assessed the certainty of evidence for three outcomes. The decrease in the frequency and intensity of bruxism (rated as “high”, even though imprecision was considered “serious”, i.e., should have been rated as “moderate”); the improvement in bruxism-related orofacial pain (rated as “very high”—a rating inexistent in the GRADE approach, that ranges from “very low” to “high”) [43]; and the decrease in bite force (rated as “very low”). The results of the certainty of evidence assessment were not used to draw conclusions, nor were they discussed, as recommended by the GRADE Working group [43].

## 3. Discussion

This overview aimed to evaluate the effectiveness of BoNT-A in managing bruxism based on existing SRs. While the included SRs [23,27,28,29,30,31,32,33,34,35,36,37,38,39] compared BoNT-A to a placebo or standard treatments—showing evidence that supports its use—the methodological quality of these reviews was generally rated as critically low according to AMSTAR-2 criteria [44]. Rather than assessing individual primary studies directly, this overview synthesizes findings from the included SRs, analyzing key outcomes, such as pain intensity, functional movement, reduction of bruxism events, and maximum bite force reduction. The discussion is centered on the conclusions drawn from these reviews, acknowledging their methodological limitations and the reliance on secondary literature.

Among the 14 SRs analyzed, only three conducted a meta-analysis to synthesize findings [28,29,39]. The remaining SRs performed a descriptive analysis of the outcomes [23,27,30,31,32,33,34,35,36,37,38]. This is understandable if we consider the heterogeneity of the studies. Regarding their population, some SRs mixed studies with other diagnoses associated with bruxism, such as temporomandibular disorder [34,35], headaches [27] and myofascial pain [27]. In terms of intervention comparisons, BoNT-A was assessed against a placebo [27,28,29,30,31,32,35,36,37,38,39], pre-intervention scores [27,29], different injection sites [30], and other alternative treatment (such as oral splinting, other medications, biofeedback and cognitive-behavioral approaches) control groups [23,28,29,31,32,33,34,35,36,37,39].

The bibliometrics showed that only two SRs were found to have registered a protocol [36,37], which may have hindered the development of work introducing bias. Booth et al. emphasized that protocol registration is crucial for enhancing transparency, preventing bias, and optimizing resources in health and social care research [45].

The overlap between the SRs was approximately 10.11%, meaning that 16 of the 47 primary studies were repeated in more than one review. The continued use of repeated studies to address the same research question may lead to research waste [46]. For instance, two SRs reached the exact same conclusion: “Infiltrations with BTX-A are a safe and effective treatment for patients with bruxism, so its use is justified in daily clinical practice, especially in patients diagnosed with severe bruxism” [31,35]. To address these issues, the publication of SRs and meta-analyses must be realigned [46]. Efforts should focus on eliminating biases and vested interests, ensuring that these reviews are better integrated with primary evidence [46]. A more rigorous and prospective approach is needed to ensure that SRs contribute meaningfully to evidence-based practice rather than diluting the field with redundant or biased information [46].

All the SRs included in this overview scored a “critically low” confidence rate, except for one that scored “low” [23], based on AMSTAR-2 [44]. These results are in line with the results of Pauletto et al. (2022) [47], which critically appraised the SRs of intervention in dentistry published between 2019 and 2020 using the AMSTAR-2 tool. The authors found that less than 1% of recently published SRs in dentistry were classified with high methodological quality, and over 50% of SRs also scored a critically low level of methodological quality [47].

Regarding the certainty of evidence, although the GRADE approach [48] has been used for almost 20 years to assess the certainty of evidence in SRs and all included SRs were published in the last 12 years, only one [37] mentioned having used it. Sendra et al. (2022) [37] applied it inconsistently and did not use it to discuss the results and draw conclusions. Therefore, no SR in this overview consistently assessed the certainty of the evidence. This emphasizes the need for systematic and transparent methods, like the GRADE approach [43], to properly assess and report the certainty of evidence in this field [49].

Only three SRs specified the method used for the bruxism assessment, with each employing a distinct approach (EMG [27], clinical diagnostic criteria [30], and PSG or EMG [23]). This variation highlights the ongoing debate regarding the most accurate and practical tools for diagnosing sleep bruxism. The lack of standardized diagnostic criteria complicates efforts to synthesize evidence from studies employing different methodologies, contributing to heterogeneity in the reported prevalence and outcomes. Addressing these discrepancies is crucial for enhancing the reliability and comparability of research findings [46], underscoring the need for a consensus on the most reliable and feasible bruxism-assessment tools. Additionally, only three SRs focused exclusively on SB [23,29,33]. The follow-up periods varied significantly across studies, contributing to the heterogeneity in reported outcomes.

Regarding the results, seven studies reported a reduction in bruxism events [29,31,32,33,34,35,38]. On the other side, De la Torre Canales (2017) [30] found that BoNT-A was effective in reducing the intensity of RMMA episodes rather than the total number of events, and Ågren et al. (2020) found this outcome to be inconclusive [27].

Also, Lobbezoo et al. stated that bruxism is primarily a disorder of central origin characterized by disturbances in the central nervous system, particularly involving the basal ganglia and neurotransmitter systems [50]. This central involvement is supported by evidence linking bruxism to sleep disturbances, dopaminergic imbalances, and responses to stress and medications [50]. Despite this central etiology, peripheral treatments, like BoNT-A injections, are employed to manage bruxism by targeting the masticatory muscles. BoNT-A works by reducing muscle activity, which can alleviate pain symptoms and provide relief from muscle-related complications, such as reduced functional movement and augmented maximum bite force. However, since bruxism originates from central mechanisms, including neural pathways and neurotransmitter disruptions, peripheral interventions might not address the underlying cause of the disorder. Consequently, while BoNT-A can mitigate the muscular manifestations and provide symptomatic relief, it may not necessarily reduce the frequency or intensity of bruxism events themselves, as the underlying central nervous system dysfunction remains unaddressed. This theoretical limitation underscores the need for a more integrated approach to bruxism treatment, one that considers both central and peripheral factors in order to achieve more comprehensive management of the disorder [50]. Additionally, many SRs do not differentiate between sleep and awake bruxism, making it difficult to determine whether the reported effects of BoNT-A apply equally to both conditions.

In terms of pain reduction, ten studies [28,29,31,32,34,35,36,37,38,39] reported a positive effect, but De la Torre Canales et al. (2017) [30] found inconclusive results. It is important to state that although BoNT-A was mentioned as a treatment for pain associated with bruxism, this condition itself does not directly cause pain. Instead, pain is primarily attributed to TMD [51]. Thus, it can be argued that the authors were effectively managing TMD with BoNT-A rather than bruxism itself. In this context, the results of these studies are consistent with the findings of De la Torre Canales et al. (2022) [52], who investigated patients with myofascial TMD-related pain and observed significant reductions in VAS values after the application of BoNT-A.

Six studies [27,28,31,34,35,38] evaluated the maximum bite force reduction, with all reporting it as effective, except for Ågren et al. (2020) [27], who found inconclusive results. Manfredini et al. (2015) mentioned the outcomes included in their article but did not clearly state in their conclusion of whether BoNT-A was able to make a difference or not [23]. More recent findings by Sitnikova et al. (2022) and Ågren et al. (2023) indicate that the reduction in the maximum bite force may last around 11 weeks [53] and up to three months [54].

Even though no included SR effectively assessed the outcome changes in functional movement, a recent study by De la Torre Canales et al. (2022) demonstrated improved lateral jaw movements and increased maximum unassisted and assisted mouth opening compared to the placebo group [55].

A notable limitation arose due to the diverse eligibility criteria across the included SRs. This heterogeneity posed challenges in synthesizing findings, as, despite the efforts to include SRs with the same populations, interventions, and outcomes, the included SRs had different eligibility criteria. Therefore, some of them have included primary studies that do not directly answer our research question. Despite this challenge, this overview provides valuable insights, though the diversity in eligibility criteria should be carefully considered when interpreting the findings.

A key limitation that future studies should address is the evolving concept of bruxism. Many of the included reviews were published before the 2018 international consensus [2], and even among the more recent ones, bruxism was often not clearly defined or distinguished from TMD. Additionally, most reviews did not incorporate the latest literature, which further differentiates between various forms of bruxism, such as AB and SB [2]. This lack of clarity in the definition and assessment impacts diagnostic accuracy and hinders a comprehensive understanding of its clinical implications.

Moreover, the common practice of assessing bruxism using a simple yes/no label—without specifying the detection method (i.e., subject-based, clinically based, or instrument-based)—is insufficient [56]. Future research should consider using the Standardized Tool for the Assessment of Bruxism (STAB), which allows for a more precise classification of AB and SB. This tool integrates self-reported symptoms, clinical evaluations, and instrumental confirmation, improving the diagnostic accuracy [57]. Furthermore, this approach helps to distinguish cases associated with underlying conditions that require targeted clinical intervention [57].

The adoption of more comprehensive diagnostic methods, beyond rigid criteria, can prevent unnecessary treatments and reinforce the role of healthcare professionals in the early detection of sleep disorders related to bruxism [56,58].

While BoNT-A injections into the masticatory muscles have been explored as a potential therapeutic option, their use is not without risk [59]. Unintended complications include paradoxical bulging, inadvertent diffusion into adjacent structures, such as the risorius muscle or parotid gland, temporary or partial facial paralysis, and bone resorption [59]. Repeated injections may lead to progressive weakening of the masticatory muscles, potentially altering the bone density and morphology [59]. These adverse effects highlight the importance of carefully weighing the risks and benefits of BoNT-A in clinical decision-making [59].

The use of BoNT-A for the management of bruxism and its associated consequences remains a topic of debate. The existing SRs are characterized by critically low methodological quality, limiting the reliability of current findings. Given these uncertainties, BoNT-A should be considered only as a last-line intervention. To establish its efficacy more definitively, further research is needed, emphasizing accurate diagnoses, standardized methodologies aligned with guidelines, such as STAB, and well-designed randomized controlled trials with larger sample sizes. Future studies should also incorporate standard follow-up periods and comprehensive outcome assessments to provide a clearer understanding of BoNT-A’s therapeutic potential. Addressing these methodological limitations is essential to ensuring patient-centered care by optimizing treatment benefits while minimizing associated risks.

## 4. Conclusions

The included SRs were scored as “critically low” and “low” quality. There is no certainty of evidence about the use of BoNT-A for bruxism, since no systematic review has consistently used the GRADE approach. There is a tendency that BTX can reduce pain, bruxism events, and the maximum bite force; however, there is no evidence to safely confirm this premise. More RCTs, with better design are necessary to clarify the benefits of BoNT-A for bruxism management.

## 5. Materials and Methods

This overview’s protocol was based on the Preferred Reporting Items for Systematic Reviews and Meta-Analyses Protocols (PRISMA-P) [60]. The protocol was registered at the Open Science Framework (OSF) platform under the number DOI: 10.17605/OSF.IO/RB45T [61]. The protocol was also previously published in the British Medical Journal Open (BMJ Open) [62].

The study adhered to chapter 22 (Overviews of reviews) of the Cochrane Handbook for Systematic Reviews of Interventions [40] and was reported following the checklist for the Preferred Reporting Items for Overviews of Reviews (PRIOR) [63] (Supplementary Material S6).

Research Question

**Figure 4:**
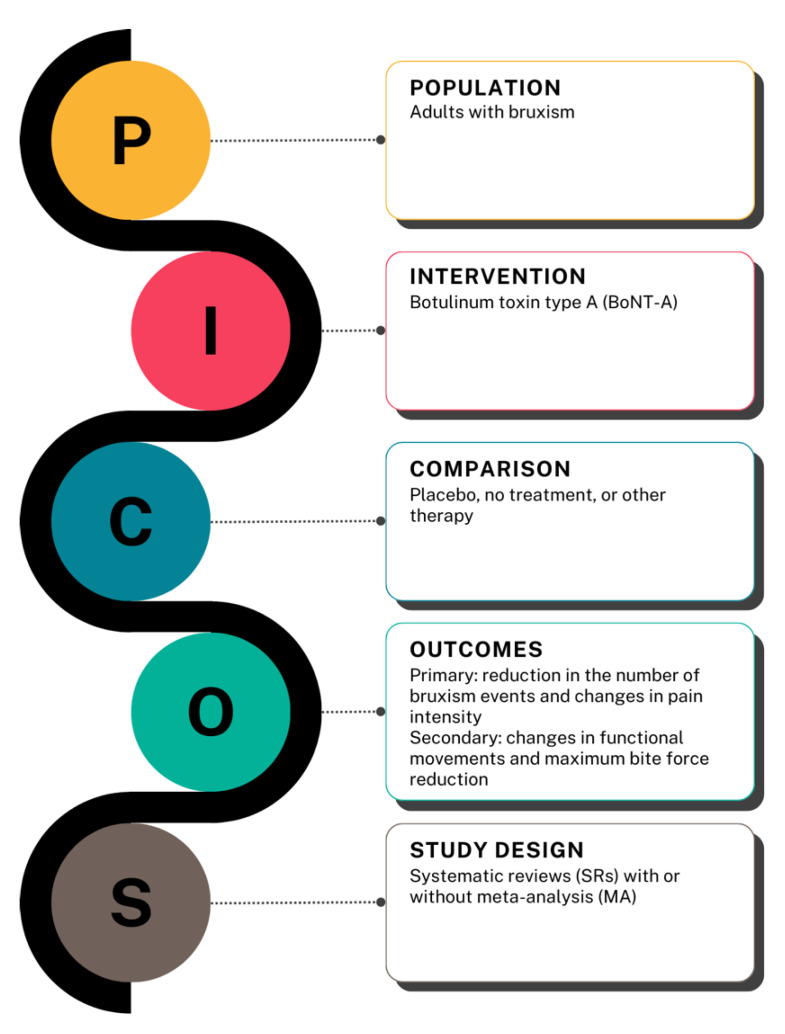
“PICOS” mnemonic as a guide to identifying the research question. Figure generated with canva.com “URL: https://www.canva.com/design/DAGW82Lt61E/7hq_zPLk2veHoC5rVeV3GA/edit?utm_content=DAGW82Lt61E&utm_campaign=designshare&utm_medium=link2&utm_source=sharebutton (accessed on 16 April 2025)”.

The “PICOS” mnemonic (Participants, Intervention, Control, Outcomes, and Study design) guided the research question. Figure 4 outlines the structuring of PICOS used in addressing the review question: “Is Botulinum Toxin Type A effective for bruxism management in adults?”.

Eligibility criteria

**Figure 5:**
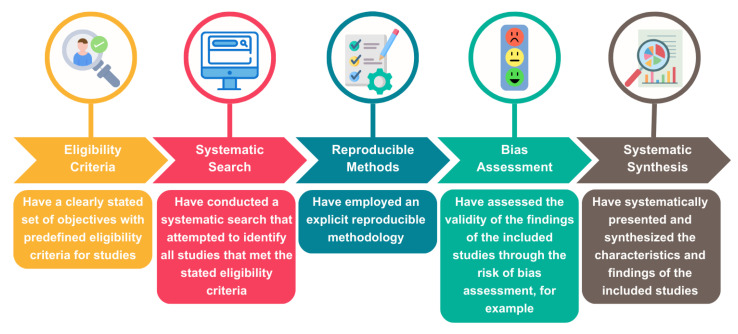
Criteria for qualifying as a systematic review (adapted from the Cochrane Handbook for Systematic Reviews of Interventions) [64]. Figure generated with canva.com “URL: https://www.canva.com/design/DAGmJWtTNOk/hHByM5vy3BkdEPMSCpcpmg/edit?utm_content=DAGmJWtTNOk&utm_campaign=designshare&utm_medium=link2&utm_source=sharebutton (accessed on 16 April 2025)”.

To be considered an SR, the study must have followed the minimum criteria outlined in Figure 5 of the Cochrane Handbook for Systematic Reviews of Interventions [40]. No time nor language restrictions were applied. SRs, with or without a meta-analysis (MA), that evaluated the efficacy of BoNT-A were included.

Participants

Only studies involving individuals aged 18 years and older diagnosed with sleep or awake bruxism were eligible for inclusion, irrespective of sex, race, ethnic background, or setting. The bruxism diagnosis had to be confirmed through self-report, clinical inspection, and/or instrumental assessment (such as polysomnography or electromyography) [1,65].

Intervention

The considered intervention was BoNT-A injections into the masseter and/or temporalis muscles.

Comparison

The intervention’s effect was assessed by comparing BoNT-A to a placebo or sham therapy, BoNT-A to no treatment, and BoNT-A to other interventions alone (e.g., occlusal splints, medications, transcutaneous electrical nerve stimulation—TENS).

Primary outcomes included the following:

Reduction of bruxism events. The number of bruxism events must have been measured via EMG and expressed as the Episodes of Rhythmic Masticatory Muscle Activity (RMMA) per hour (N) and/or the duration of these RMMA episodes.

Changes in pain intensity. The pain intensity must have been measured using a Visual Analog Scale (VAS) or another objective, validated instrument. If more than one scale was used, VAS results were prioritized for the analysis.

Secondary outcomes included the following:

Change in functional movement. Range of motion and changes in function (jaw movements) must have been assessed using objective measures (measured using a ruler or a caliper, expressing the range of motion in millimeters or centimeters) for the following movements: maximum mouth opening (passive and active); lateral movement (left and right); protrusive movement.

Maximum bite force reduction. Maximum occlusal force (kg) and maximum bite force (kg) must have been measured using objective measures or validated tests.

The exclusion criteria were as follows:

- SRs that included participants outside the adult age range;
- SRs that included cases of bruxism caused by or associated with neurological disorders;
- SR that included participants using BoNT-A therapy for conditions other than bruxism, such as the management of temporomandibular disorders or the use for aesthetic purposes;
- Exclusion of publication types other than SRs, such as primary studies, editorials, letters to the editor, case reports, conference papers and proceedings, book chapters, preprints, and patents.

Information sources

The search was applied to seven databases (Cochrane Library, Embase, LILACS, Livivo, MEDLINE via PubMed, Scopus, and Web of Science). Additionally, a search for grey literature was conducted on Google Scholar and ProQuest Dissertations and Theses Global. Hand searches of bibliographies from the included studies and key journals were conducted. Experts were also consulted to identify additional relevant studies.

Search strategy

With the assistance of an experienced health sciences librarian, search strategies were developed according to the specificities of each database and grey literature source. The Supplementary Material S7 contains the detailed search strategies. The electronic search was carried out on 16 June 2024. No time or language restrictions were applied.

Selection process

The identified references were imported into a reference software manager (EndNote X9^®^; Thomson Reuters, Philadelphia, PA, USA) [66], where the duplicates were removed. A unique file was then exported to Rayyan^®^ Online Software (Qatar Computing Research Institute, Ar-Rayyan, Qatar) [67], where two independent reviewers (MSC and JMOD) conducted an initial screening based on titles and abstracts (phase 1), followed by a full-text assessment (phase 2). Disagreements at any phase were resolved with input from a third reviewer (HP).

Data collection process

Data from the selected articles were initially collected by one independent author (MSC). This information was then verified by a second author (JMDO). Any discrepancies between the authors were discussed and resolved in a consensus meeting. If essential data for this overview were missing or unclear, attempts were made to contact the study’s corresponding author for clarification. If there was no response after three attempts within three weeks, the data were noted as “data missing or unclear”.

Data items

The following variables were extracted and organized in tables: study characteristics (author, country, year); objectives; participants’ characteristics (number, age, description of the condition (bruxism), and criteria for bruxism diagnosis); intervention and comparison (site, drug, follow-up); results for each outcome; conclusions; funding; conflict of interest; protocol registry; searched databases; additional literature search; number of randomized controlled trials (RCTs) and/or non-RCTs included; risk of bias assessment tools; MA development; use of the Grading of Recommendations Assessment, Development and Evaluation (GRADE) approach; compatibility with a Cochrane review; publication bias.

Methodological quality

The methodological quality of the included SRs was assessed based on the AMSTAR-2 (A MeaSurement Tool to Assess systematic Reviews) checklist [44] by two independent authors (MSC and JMOD). The checklist comprised 16 questions, seven of which are deemed critical. Each question is answered as “yes”, “partial yes”, or “no”, and these responses determined the overall confidence level of the reviews as “high”, “moderate”, “low”, or “critically low” [44,47].

Synthesis methods

A narrative synthesis was used to formally combine the data from individual SRs. The results were presented using color charts and figures. To address possible data overlap from duplicated primary studies across SRs, the overlap was quantified using the corrected covered area and visualized through the Graphical Representation of Overlap for OVErviews (GROOVE) tool [40,41,42], presented in Supplementary Material S8.

Certainty assessment

The process of assessing the certainty, or confidence, in the body of evidence for an outcome using the GRADE approach [43] or other was analyzed for each included SR. For those using the GRADE approach [43], the following information was sought: considered outcomes; GRADE key domains responsible for downgrading; study limitations (risk of bias), inconsistency, indirectness, imprecision, and publication bias) with justification; and certainty level per outcome (high, moderate, low, or very low). The GRADE approach was not assessed in an SR that did not report it.

## 6. Strengths and Limitations of This Study

The researchers followed chapter 22 (Overviews of reviews) of the Cochrane Handbook for Systematic Reviews of Interventions and used the checklist for the Preferred Reporting Items for Overviews of Reviews (PRIOR) to guide the structure of their manuscript. A comprehensive literature search encompassed seven databases, grey literature, references from included studies, and consultations with field experts. A health sciences librarian contributed to refining the search strategy. The screening of abstracts and full texts involved multiple reviewers to maximize the identification of relevant studies. Methodological quality assessment employed AMSTAR-2 for the included SRs. However, the overview was challenged by the diverse eligibility criteria among the SRs included, highlighting a limitation of the study. Additionally, as the methodology focused exclusively on published SRs, primary studies, books, and other types of publications were not considered.

## 7. Protocol Alterations

The title of this overview was updated from “Botulinum toxin for the management of bruxism: an overview of review” to “Botulinum toxin for bruxism: an overview”. The research question was rephrased from “What is the current knowledge regarding the clinical effectiveness of BoNT-A for bruxism control compared to placebo, no treatment, or other treatments in adults?” to “Is Botulinum Toxin Type A (BoNT-A) effective for bruxism management in adults?”. No study selection criteria based on context (e.g., “Studies carried out in dental or health research centers will be included”) were applied. The explanation in the inclusion criteria section regarding the units of measurement used to evaluate the outcomes was restructured. No assessment of measures of effect was conducted. As it was not possible to obtain a second author to collect data, one reviewer undertook data collection while another cross-checked the gathered information. The risk of bias was only assessed using AMSTAR-2, and the Risk of Bias Assessment Tool for Systematic Reviews (ROBIS) was not used. No statistical analysis was possible due to study heterogeneity. The GRADE approach was not assessed in an SR that did not report it.

## Data Availability

The original contributions presented in this study are included in the article/Supplementary Materials. Further inquiries can be directed to the corresponding author.

## Supplementary Materials

The following supporting information can be downloaded at: https://www.mdpi.com/article/10.3390/toxins17050249/s1, Supplementary Material S1: Excluded articles with reasons (*n* = 16); Supplementary Material S2: PICOS analysis (*n* = 14); Supplementary Material S3: Descriptive overview of included systematic reviews (*n* = 14); Supplementary Material S4: Bibliometric characteristics (*n* = 14); Supplementary Material S5: Matrix of evidence with included studies per included systematic review; Supplementary Material S6: Checklist for the Preferred Reporting Items for Overviews of Reviews (PRIOR); Supplementary Material S7: Search strategies; Supplementary Material S8: Graphical Representation of Overlap for OVErviews (GROOVE) Tool.

## Abbreviations

The following abbreviations are used in this manuscript:

AB	Awake Bruxism
AMSTAR-2	A MeaSurement Tool to Assess systematic Reviews
BoNT-A	Botulinum Toxin Type A
BTX-A	Botulinum Toxin Type A
CCA	Corrected Covered Area
COBE	Brazilian Centre for Evidence-Based Research
DC/TMD	Diagnostic Criteria for Temporomandibular Disorders
EMG	Electromyography
GRADE	Grading of Recommendations, Assessment, Development, and Evaluation
GROOVE	Graphical Representation of Overlap for OVErviews
MA	Meta-Analysis
OSF	Open Science Framework
PICOS	Participants, Intervention, Control, Outcomes, and Study design
PRIOR	Preferred Reporting Items for Overviews of Reviews
PRISMA-P	Preferred Reporting Items for Systematic Reviews and Meta-Analyses Protocols
PSG	Polysomnography
RCT	Randomized Controlled Trial
ROBIS	Risk of Bias in Systematic Reviews
RMMA	Rhythmic Masticatory Muscle Activity
SB	Sleep Bruxism
SR	Systematic Review
STAB	Standardized Tool for the Assessment of Bruxism
TMD	Temporomandibular Disorder
VAS	Visual Analog Scale
