# Supplementary material for: Botulinum Toxin for Bruxism: An Overview
Source: Toxins (Basel). 2025 May 16;17(5):249. doi: 10.3390/toxins17050249 (PMC12115368; doi:10.3390/toxins17050249)
Supplement: Supplementary file 1 [file toxins-17-00249-s001.zip › toxins-3487049-supplementary.pdf]

# Supplementary Materials: Botulinum Toxin for Bruxism: An Overview

**Manuella Salm Coelho, Júlia Meller Dias de Oliveira, Helena Polmann, Patrícia Pauletto, Cristine Miron Stefani, Lara Catarine De Luca Maciel and Graziela De Luca Canto**

*Supplementary Material S1.* Excluded articles with reasons (n=16).

| *Study* | *Reason for exclusion* |
| --- | --- |
| *Anandan & Jankovic (2021)* | Review did not meet the minimum criteria for SRs |
| *Balanta-Melo et al. (2022)* | Review did not meet the minimum criteria for SRs |
| *Bardavid (2022)* | Co-intervention |
| *Buzatu et al. (2024)* | Study not directly related to the efficacy of botulinum toxin injection in adults with primary bruxism |
| *Cunha et al. (2022)* | Review did not meet the minimum criteria for SRs |
| *De Lima et al. (2020)* | Review did not meet the minimum criteria for SRs |
| *Dimartini et al. (2022)* | Review did not meet the minimum criteria for SRs |
| *Ferreira et al. (2023)* | Review did not meet the minimum criteria for SRs |
| *Khawja et al. (2024)* | Study not directly related to the efficacy of botulinum toxin injection in adults with primary bruxism |
| *Kumar & Spivakovsky (2018)* | Review did not meet the minimum criteria for SRs |
| *Macedo et al. (2007)* | Study not directly related to the efficacy of botulinum toxin injection in adults with primary bruxism |
| *Minakuchi et al. (2022)* | Co-intervention |
| *Sierra Betancourth et al. (2022)* | Review did not meet the minimum criteria for SRs |
| *Silva et al. (2022)* | Review did not meet the minimum criteria for SRs |
| *Teixeira & Sposito (2013)* | Review did not meet the minimum criteria for SRs |
| *Tinastepe et al. (2015)* | Review did not meet the minimum criteria for SRs |

*Supplementary Material S2.* PICOS analysis (n = 14).

| *Study* | *Population (N)* | *Bruxism diagnosis method* | *Interventions and comparators* | *Outcome(s)* | *Follow up range* | *Primary studies* |
|---|---|---|---|---|---|---|
| *Ågren et al. (2019)* | "Study groups treated for headache, bruxism, myofascial pain or masseter hypertrophy" (N=83) | EMG | Intervention: BT injections into the masseter and/or temporal muscles<br>Comparison: "Pre-injection values and/or placebo injections" | "Difference in EMG activity or change in bite force" | 14d - 6mos | RCT (4)<br>nRCT (0) |
| *Chen et al. (2023)* | Adult patientes with primary bruxism (N=383) | MD | BT injections into the masseter and/or temporal muscles<br>Placebo; no injection; conventional treatment (splinting) | Maximal bit force (kg); maximal mouth opening (mm); masseter thickness (mm); and pain (VAS) | 6mos -12mos | RCT (7)<br>nRCT (3) |
| *Cheng et al. (2022)* | Adults diagnosed with bruxism (N=148) | MD | BT injections into the masseter and/or temporal muscles<br>Placebo; before-injection; and "other interventional approaches, such as cognitive-behavioral approaches" | Pain at rest; pain at chewing; frequency of bruxism events; self-assessment by patients; and safety of BTA | 2wks - 6mos | RCT (6)<br>nRCT (0) |
| *De La Torre Canales et al. (2017)* | "Bruxism patients" (N=188) | Screening-oriented clinical diagnostic criteria | BT injections into the masseter and/or temporal muscles<br>No control group; placebo; comparision on different injection sites | Perception of treatment efficacy; pain at chewing; number of bruxism events during sleep; peak amplitude of EMG burst of RMMA episodes; frequency, number of bursts or duration of RMMA episodes | None or 12wks - 6mos | RCT (3)<br>nRCT (2) |

*Supplementary Material S2.* PICOS analysis (n = 14). (Continued)

| *Study* | *Population (N)* | *Bruxism diagnosis method* | *Interventions and comparators* | *Outcome(s)* | *Follow up range* | *Primary studies* |
|---|---|---|---|---|---|---|
| *Fernández-Núñez et al. (2019)* | "Bruxist patients older than 18 years which the effect of botulinum toxin in the treatment of bruxism compared with traditional therapy is analyzed" (N=112) | MD | BT injections into the masseter and/or temporal muscles<br>Placebo or "the use of traditional methods for the treatment of bruxism. such as occlusal splints, other medications or cognitive-behavioral therapy" | Bruxism events (PSG); painful symptoms (subjective questionnaire or VAS); jaw movements (mm); duration of biting and opening (T-Scan); maximum occlusal force (T-Scan); and symmetrical distribution of occlusal force (T-Scan) | 1wk - 1y | RCT (4)<br>nRCT (0) |
| *Long et al. (2012)* | Bruxism patients over 18 years old (N=57) | MD | BT injections into the masseter and/or temporal muscles<br>"Placebo or other interventional procedures" | Frequency of bruxism events (EMG); pain (VAS); adverse effects; and sleep quality improvement | 1wk - 6mos | RCT (2)<br>nRCT (2) |
| *Manfredini et al. (2015)* | Adults with bruxism (N=233) | PSG or sleep-time EMG of the masticatory muscles | BT injections into the masseter and/or temporal muscles<br>Oral appliances, pharmacological approaches (including botulinum toxin, clonazepam, and clonidine), biofeedback, and cognitive behavioral approaches; injections into both the masseter and temporalis muscles | Frequency, number of bursts, or duration for RMMA episodes; and peak amplitude of EMG burst of RMMA episodes in the injected muscles | Baseline - 12wks | RCT (12)<br>nRCT (2) |

*Supplementary Material S2.* PICOS analysis (n = 14). (Continued)

| *Study* | *Population (N)* | *Bruxism diagnosis method* | *Interventions and comparators* | *Outcome(s)* | *Follow up range* | *Primary studies* |
|---|---|---|---|---|---|---|
| *Miron et al. (2022)* | Adults over 18 years nocturnal bruxism (N=61) | MD | BT injections into the masseter and/or temporal muscles<br>Pramipexole (PPX); rabeprazole; biofeedback splitters (BFB); oclussal splints(AOS); sleep hygiene tips and static muscle stretching exercises with a stretching device; Massage; mandibular advancement device (MAD); maxillary occlusal splints (MOS) | Contraction intensity reduction, total sleep time improvement, reduction on number and duration of bruxism events; sleep bruxism intensity or severy reduction; facial pain reduction; increase maximum mouth opening; quality of life improvement | 12wk | RCT (9)<br>nRCT (0) |
| *Patel et al. (2019)* | Patients of any age that have TMD of myogenous nature and/or bruxism (N=324) | MD | BT injections into the masseter and/or temporal muscles<br>Pain; maximum mouth opening; occlusal force; EMG reading at rest and maximum clenching; bruxism events during sleep | Pain; frequency of bruxism events; maximum mouth opening; occlusal force; and EGM readings of muscles of mastication | Immediat pos-op - 6mos | RCT (11)<br>nRCT (0) |
| *Rajamoorthy et al. (2023)* | Individuals with Temporomandibular Disorders and/or Bruxism (N=189) | MD | BT injections into the masseter and/or temporal muscles<br>Traditional methods | Pain (VAS) | Baseline - 1y | RCT (6)<br>nRCT (0) |
| *Sendra et al. (2021)* | "Human adults with primary bruxism" (N=314) | MD | BT injections into the masseter and/or temporal muscles<br>Placebo, no injections, or other treatments | "Improvement of symptoms" | Baseline - 1y | RCT (6)<br>nRCT (4) |

*Supplementary Material S2.* PICOS analysis (n = 14). (Continued)

| *Study* | *Population (N)* | *Bruxism diagnosis method* | *Interventions and comparators* | *Outcome(s)* | *Follow up range* | *Primary studies* |
|---|---|---|---|---|---|---|
| *Sendra et al. (2022)* | "Adult humans with primary bruxism" (N= 364) | MD | BT injections into the masseter and/or temporal muscles<br>Placebo or other treatments without injections | "Improvement of bruxism symptom occurrences" | Initial - 1y | RCT (11)<br>nRCT (0) |
| *Sposito et al. (2014)* | Adults with bruxism (N=32) | MD | BT injections into the masseter and/or temporal muscles<br>Placebo | Frequency of bruxism; pain at chewing | 1wk - 6mo | RCT (2)<br>nRCT (0) |
| *Zhang et al. (2023)* | Humans (N=365) | MD | BT injections into the masseter and/or temporal muscles<br>Placebo and oral splinting | Pain (VAS) | 6mos - 1y | RCT (11)<br>nRCT (0) |

BT (Botulinum Toxin); BTA (Botulinum Toxin Type A); BTX (Botulinum Toxin A); d (days); EMG (electromyography); GRADE (Grading of Recommendations Assessment, Development and Evaluation); MA (Meta-analysis); MD (Missing Data); mm (millimeters); mo (month); N (number of patients); NA (Non Applicable); nRCT (Non-Randomized Controlled Trial); placebo (saline solution injection); PROSPERO (International Prospective Register of Systematic Reviews); PSG (polysomnography); RCT (Randomized Controlled Trial); RMMA (Rhythmic Masticatory Muscle Activity); RoB (Risk of Bias); SR (Systematic Review); TMD (Temporomandibular Dysfunction); BTX (Botulinum Toxin); VAS (Visual Analog Scale); wk (week); y (year)

*Supplementary Material S3.* Descriptive overview of included systematic reviews (n=14).

| *Study* | *Objective(s)* | *Results* | *Conclusion* |
|---|---|---|---|
| *Ågren et al. (2019)* | Evaluate relevant evidence and "assess whether BTA injections influence bruxism and result in fewer episodes of clenching or less bite force and if this effect is long-lasting" | "3 articles measured EMG and 1 bite force. 1 article did not record a significant drop of activity, 1 article recorded reduction midway and at final endpoint. 2 articles recorded initial reduction, but a non-significant difference at later follow up" | "The available research is inconclusive and does not show enough evidence that bruxism can be treated with BTA injections. However, promising results have been shown in individual studies and further research in this area is needed" |
| *Chen et al. (2023)* | Compile evidence on BTA for bruxism management efficacy | The maximal biting force declined significantly one to three months post BTA injections versus oral splints and saline injections. Between three and six months, the BTA group showed notably higher biting strength than the oral splinting group, with no significant difference from the saline group. Pain reduction post-botulinum treatment followed a similar trend. Each unit increase in BTA dosage correlated with a 0.0831-point decrease in pain severity | "BTA injections effectively manage bruxism, with effects seen in less than a month and lasting up to 24 weeks, although occlusal splints offer longer-lasting benefits. Higher BTA doses correlate with better pain relief, making it suitable for bruxers having trouble with splint adherence or seeking early symptom improvement" |
| *Cheng et al. (2022)* | "Explore the treatment efficacy of botulinum-A in nocturnal bruxism" | "Compared with the placebo group, the BTA group showed that it significantly improved the pain at rest index scores, slightly improved the pain at chewing index scores, and the number of bruxism events were significantly decreased in the before-injection group compared with that in the after-injection group" | "The results of this study suggest that BTA possesses significant therapeutic efficiency for the relief of pain and events of bruxism. However, whether the events of bruxism would recur or rebound after botulinum toxin injection needs more follow-up clinical evidence" |
| *De La Torre Canales et al. (2017)* | "Assessing the effects of botulinum toxin injections in the management of bruxism" | The studies exhibited methodological variability. While subjective assessments consistently showed improvements in pain and jaw stiffness, objective assessments yielded mixed results, with one study reporting reduced bruxism episodes in masseter muscles but not in temporalis muscles, while another found no reduction in bruxism episodes after BTA injection. Quality assessment indicated varying levels of bias and methodological rigor across the studies | "Despite the paucity of works on the topic, BTA seems to be a possible management option for sleep bruxism, minimizing symptoms and reducing the intensity of muscle contractions, although further studies are necessary especially as far as the treatment indications for bruxism itself is concerned" |

*Supplementary Material S3.* Descriptive overview of included systematic reviews (n=14). (Continued)

| *Study* | *Objective(s)* | *Results* | *Conclusion* |
|---|---|---|---|
| *Fernández-Núñez et al. (2019)* | Determine if, in bruxist patients, the use of BTA is more effective than the traditional methods used up to now on the signs and symptoms of bruxism | "BTA injections can reduce the frequency of bruxism episodes, decrease pain levels and maximum occlusal force generated by this pathology, offer superior efficacy in the treatment of bruxism compared to control groups who were treated with placebo or with traditional methods for the treatment of bruxism" | "Infiltrations with BTA are a safe and effective treatment for patients with bruxism, so its use is justified in daily clinical practice, especially in patients diagnosed with severe bruxism" |
| *Long et al. (2012)* | "Assessing the efficacy of BTs on bruxism" | BT injections effectively decrease bruxism frequency and pain, satisfying patient assessments of their effectiveness. Studies show their comparable efficacy to oral splints. Additionally, doses below 100 units are safe for healthy patients, affirming BT injections as both effective and safe for bruxism management | BT injections as both effective and safe for bruxism management |
| *Manfredini et al. (2015)* | "Update the bruxism management review published by Lobbezoo et al.in 2008, by assessing the most recent literature on the topic" | Pharmacological management studies, involving 90 subjects, suggest botulinum toxin injections reduce SB episode intensity but not frequency | "There is not enough evidence to define a standard of reference approach for SB treatment, except for the use of OA. Future studies on the indications for SB treatment are recommended" |
| *Miron et al. (2022)* | Conduct a systematic review of the therapeutic approaches of nocturnal bruxism | BTA decreases bruxism episodes and total bruxism time | "The physical methods of therapy can reduce the number of bruxism events, their duration, and may alleviate the pain of the masticatory muscles. Pharmacotherapy has proven low efficiency. Botulinum toxin type A is an effective therapeutic option for controlling the nocturnal bruxism" |

*Supplementary Material S3.* Descriptive overview of included systematic reviews (n=14). (Continued)

| *Study* | *Objective(s)* | *Results* | *Conclusion* |
|---|---|---|---|
| *Patel et al. (2019)* | "To establish the usefulness of BTA when treating patients with TMD and/or bruxism, and thereby determine whether there may be an appropriate purpose for the prescription of BTA in the management of these patients" | Studies consistently show pain score reductions in BT groups compared to controls, lasting up to three months. Secondary outcomes, like maximum mouth opening, vary across studies, with improvements seen in some and reductions in others | The evidence for BT in managing TMD and/or bruxism is not entirely conclusive, though promising results in several studies warrant further investigation. While BT should be considered, its financial implications and potential side effects suggest that conservative options like self-management and physical therapies should be explored first |
| *Rajamoorthy et al. (2023)* | Analyze the existing literature on the use of BTA injections into Facial Muscles for the treatment of Temporomandibular Disorders and Bruxism | "Studies show that BTX-A injections can reduce the frequency of bruxism episodes, decrease pain levels and maximum occlusal force generated by this pathology, offer superior efficacy in the treatment of bruxism compared to control groups who were treated with placebo or with traditional methods for the treatment of bruxism" | BTA infiltrations effectively reduce the frequency of bruxism episodes, masticatory force, and associated pain levels, enhancing patients' quality of life. At doses below 100UI, it's safe with minimal adverse effects in healthy individuals. BTA emerges as a safe and efficient treatment surpassing traditional methods like occlusal splints or drugs, justifying its use in clinical practice, particularly for severe bruxism cases |
| *Sendra et al. (2021)* | Examine the clinical effects of BTA injections for treating primary bruxism in adults | The studies included 8-120 participants and utilized four brands of BT, ranging from 14 to 200 units per participant. Follow-up periods varied from 1 week to 15 months, employing different clinical evaluation methods. Pain assessment after 6 months utilized the visual analog scale (VAS) in two articles, with one using a 0-5 scale and the other a 0-10 scale. MA was not feasible due to study heterogeneity. Risk of bias assessments using the Fowkes and Fulton checklist revealed six studies with low risk, moderate risk for one, and high risk for another. All studies supported the efficacy and safety of BT injections in reducing primary bruxism symptoms. Out of 237 participants undergoing BT treatments, only 5 experienced short-term adverse effects | BTA injections are effective in the treatment of the symptoms of primary bruxism in adults. |

*Supplementary Material S3.* Descriptive overview of included systematic reviews (n=14). (Continued)

| *Study* | *Objective(s)* | *Results* | *Conclusion* |
|---|---|---|---|
| *Sendra et al. (2022)* | "Analyze the clinical outcomes of BTA in the management of primary bruxism in adults" | With sample sizes ranging from 12 to 94 participants. Various brands of BTA were used, with doses between 40 to 200 units per participant, and follow-up periods ranging from 1 week to 1 year. GRADE methodology favored this intervention. Among 211 participants receiving BTA treatments, only 5 reported transient adverse effects, including mastication difficulty in three cases and changes in smile appearance in 12 and 2 cases respectively. Due to study heterogeneity, meta-analysis was not feasible | All studies reviewed confirm the efficacy of BTA injections in alleviating bruxism symptoms, even with doses below 25U targeted solely at the masseter muscles |
| *Sposito et al. (2014)* | "Systematize scientific evidence on the effectiveness of BTA in the treatment of bruxism" | "Two studies of double blind randomized clinical trials were selected. The two clinical studies showed that the application of botulinum toxin could diminish levels of pain, lower the frequency of occurrences of bruxism, and satisfy the patients in terms of efficacy of the botulinum toxin in this pathology, in addition to having no important adverse effects. Thus, the treatment with BTA could present itself as one possible treatment for patients with bruxism" | "More studies are needed that follow the quality criteria to reach a definitive conclusion about efficacy and safety" |
| *Zhang et al. (2023)* | Estimate the efficiency of BTA injection in relieving pain caused by bruxism at different follow-up periods | Ten studies analyzed BTA's effectiveness in reducing bruxism-related pain using VAS scores at 1st, 3rd, and 6th-month follow-ups. A forest plot of 26 records indicated a mean reduction of 4.06 (95% CI, 3.37 to 4.75) in VAS scores post-injection, with significant reductions observed across all follow-up periods. In contrast, the Bayesian meta-analysis of seven RCTs favored BTA over oral splinting or saline placebo at the 3rd-month follow-up, with a probability plot indicating BTA as the most effective approach at any follow-up period, demonstrating reliable convergence after 40,000 iterations | BTA significantly relieves the pain of bruxism for 6 months after injection, and its therapeutic efficacy was higher than that of oral splinting. Nevertheless, further long-term follow-up randomized controlled trials comparing BTA with other management or drugs are warranted |

BT (Botulinum Toxin); BTA (Botulinum Toxin Type A); BTX (Botulinum Toxin A); d (days); EMG (electromyography); GRADE (Grading of Recommendations Assessment, Development and Evaluation); MA (Meta-analysis); MD (Missing Data); mm (millimeters); mo (month); N (number of patients); NA (Non Applicable); nRCT (Non-Randomized Controlled Trial); placebo (saline solution injection); PROSPERO (International Prospective Register of Systematic Reviews); PSG (polysomnography);

RCT (Randomized Controlled Trial); RMMA (Rhythmic Masticatory Muscle Activity); RoB (Risk of Bias); SR (Systematic Review); TMD (Temporomandibular Dysfunction); BTX (Botulinum Toxin); VAS (Visual Analog Scale); wk (week); y (year)

*Supplementary Material S4.* Bibliometric characteristics (n=14).

| *Author (year)* | *Country of the authors* | *1. Cochrane review 2. GRADE 3. Meta-analysis 4. Protocol registration* | *Database(s) searched* | *1.Grey literature 2.Experts 3.Reference Lists* | *Risk of bias assessment tool* | *Software used in the MA* | *Number of studies included in the MA* | *Conflict of Interest* |
|---|---|---|---|---|---|---|---|---|
| *Ågren et al. (2019)* | Sweden | 1.No 2.No 3.No 4.No | PubMed, Web of Science, Scopus, Ovid, and EBSCO | 1.No 2.No 3.Yes | RCT: Jadad Scale nRCT: NA | NA | NA | No |
| *Chen et al. (2023)* | Taiwan | 1.No 2.No 3.Yes 4.No | PubMed, Embase, and Scopus | 1.No 2.No 3.Yes | RCT: RoB 2 nRCT: ROBINS I | Review Manager (version 5.4; The Cochrane Collaboration, London, United Kingdom) | 7 | Unclear |
| *Cheng et al. (2022)* | Republic of China | 1.No 2.No 3.Yes 4.No | PubMed, Web of Science, Cochrane, Embase, and Clinical Trials | 1.No 2.No 3.Yes | RCT: Cochrane Collaboration Tool for Assessing RoB in Randomized Trials nRCT: NA | Review Manager (version 5.3; The Cochrane Collaboration, London, United Kingdom) | 4 | No |

*Supplementary Material S4.* Bibliometric characteristics. (Continued)

| *Author (year)* | *Country of the authors* | *1. Cochrane review 2. GRADE 3. Meta-analysis 4. Protocol registration* | *Database(s) searched* | *1.Grey literature 2.Experts 3.Reference Lists* | *Risk of bias assessment tool* | *Software used in the MA* | *Number of studies included in the MA* | *Conflict of Interest* |
|---|---|---|---|---|---|---|---|---|
| *De La Torre Canales et al. (2017)* | Brazil | 1.No<br>2.No<br>3.No<br>4.No | PubMed , Scopus, Web of Science, Embase, Cochrane, Scielo, and Lilacs | 1.No<br>2.No<br>3.No | RCT: Cochrane Collaboration Tool for Assessing RoB in Randomized Trials<br>nRCT: Critical Appraisal Skills Programme (CASP) Before–After Study Checklist | NA | NA | No |
| *Fernández-Núñez et al. (2019)* | Spain | 1.No<br>2.No<br>3.No<br>4.No | PubMed, Cochrane Library, and Scopus | 1.No<br>2.No<br>3.No | RCT: Cochrane Criteria for the Risk Assessment of Bias (version 5.1.0)<br>nRCT: NA | NA | NA | No |
| *Long et al. (2012)* | Republic of China | 1.No<br>2.No<br>3.No<br>4.No | PubMed, Embase, Science Citation Index and Cochrane Central Register of Controlled Trials, and Clinical Trials | 1.Yes (SIGLE)<br>2.No<br>3.No | RCT: Cochrane Handbook<br>nRCT: Cochrane Reviewers' Handbook | NA | NA | No |

*Supplementary Material S4.* Bibliometric characteristics. (Continued)

| *Author (year)* | *Country of the authors* | *1. Cochrane review 2. GRADE 3. Meta-analysis 4. Protocol registration* | *Database(s) searched* | *1.Grey literature 2.Experts 3.Reference Lists* | *Risk of bias assessment tool* | *Software used in the MA* | *Number of studies included in the MA* | *Conflict of Interest* |
|---|---|---|---|---|---|---|---|---|
| *Manfredini et al. (2015)* | Italy | 1.No 2.No 3.No 4.No | Medline and Scopus | 1.Yes (Google Scholar) 2.No 3.No | RCT: Cochrane Collaboration Tool for Assessing RoB in Randomized Trials nRCT: Critical Appraisal Skills Programme (CASP) Cohort Study Checklist | NA | NA | No |
| *Miron et al. (2022)* | Romania | 1.Yes 2.No 3.No 4.No | PubMed and ScienceDirect | 1.Yes (Google Scholar) 2.No 3.No | RCT: Jadad Scale nRCT: NA | NA | NA | No |
| *Patel et al. (2019)* | United Kingdom | 1.No 2.No 3.No 4.No | Medline, Embase, PubMed, and Cochrane Central Register of Controlled Trials | 1.Yes (SIGLE) 2.No 3.No | RCT: Cochrane Handbook nRCT: NA | NA | NA | Unclear |
| *Rajamoorthy et al. (2023)* | India | 1.No 2.No 3.No 4.No | PubMed, Cochrane Library, and LILAC | 1.Yes (Google Scholar) 2.No 3.No | RCT: Cochrane Handbook nRCT: NA | NA | NA | Unclear |

*Supplementary Material S4.* Bibliometric characteristics. (Continued)

| *Author (year)* | *Country of the authors* | *1. Cochrane review 2. GRADE 3. Meta-analysis 4. Protocol registration* | *Database(s) searched* | *1.Grey literature 2.Experts 3.Reference Lists* | *Risk of bias assessment tool* | *Software used in the MA* | *Number of studies included in the MA* | *Conflict of Interest* |
|---|---|---|---|---|---|---|---|---|
| *Sendra et al. (2021)* | Brazil | 1.No 2.No 3.No 4.Yes. PROSPERO | PubMed, Web of Science, Scopus, LILIACS, and Cochrane Library | 1.Yes (Open Gray) 2.No 3.No | RCT: Fowkes and Fulton checklist nRCT: Fowkes and Fulton checklist | NA | NA | Unclear |
| *Sendra et al. (2022)* | Brazil | 1.No 2.Yes 3.No 4.Yes. PROSPERO | PubMed, Web of Science, Scopus, LILACS, and Cochrane Library | 1.Yes (Open Gray) 2.No 3.No | RCT: RoB 2 nRCT: NA | NA | NA | Unclear |
| *Sposito et al. (2014)* | Brazil | 1.No 2.No 3.No 4.No | PubMed and Allergan Product Literature | 1.No 2.No 3.No | RCT: Jadad Scale nRCT: NA | NA | NA | Unclear |
| *Zhang et al. (2023)* | Republic of China | 1.No 2.No 3.Yes 4.No | Web of Science, PubMed, Embase, and Cochrane Library | 1.No 2.No 3.No | RCT: Cochrane Handbook nRCT: The nRCT of Interventions tool | R version 4.2.0 | 7 | No |

RCT (Randomized Controlled Trial); nRCT (Non-Randomized Controlled Trial); NA (Not Applicable); GRADE (Grading of Recommendations Assessment Development and Evaluation); MA (Meta-analysis); PROSPERO (International Prospective Register of Systematic Reviews); RoB (Risk of Bias); ROBINS-I (Risk Of Bias In Non-Randomized Studies of Interventions); CASP (Critical Appraisal Skills Programme); SIGLE (System for Information on Grey Literature in Europe); LILACS (Latin American and Caribbean Health Sciences Literature).

*Supplementary Material S5.* Matrix of evidence with included studies per included systematic review.

| *MATRIX OF EVIDENCE* | | | | | | | | | | | | | |
|---|---|---|---|---|---|---|---|---|---|---|---|---|---|
| *Primary Studies* | *Systematic Reviews* | | | | | | | | | | | | |
| *Study ID* | *Ågren et al.(2020)* | *Chen et al. (2023)* | *Cheng et al. (2022)* | *De la Torre Canales et al. (2017)* | *Fernández -Núñez et al. (2019)* | *Long et al. (2012)* | *Manfredini et al. (2015)* | *Miron et al. (2022)* | *Patel et al. (2019)* | *Rajamoorthy et al. (2023)* | *Sendra et al. (2021) & Sendra et al. (2022)* | *Sposito et al. (2014)* | *Zhang et al. (2024)* |
| Abekura et al. (2008) | | | | | | | 1 | | | | | | |
| Ali et al. (2021) | | | | | | | | | | | 1 | | |
| Al-Wayli et al. (2017) | | 1 | 1 | | 1 | | | | | 1 | 1 | | 1 |
| Al-Wayli et al. (2024) | | 1 | | | | | | | | | 1 | | 1 |
| Arima et al. (2012) | | | | | | | 1 | | | | | | |
| Asutay et al. (2017) | | 1 | | | | | | | | | | | 1 |
| Baughman et al. (2014) | | | | | | | | | | 1 | | | |
| Bergmann et al. (2020) | | | | | | | | 1 | | | | | |
| Bolayir et al. (2005) | | | | 1 | | 1 | | | | | | | |
| Carli et al. (2016) | | | | | | | | | 1 | | | | |
| Carra et al. (2010) | | | | | | | 1 | | | | | | |
| Cahlin et al. (2016) | | | | | | | | 1 | | | | | |
| Chaurand et al. (2017) | | | | | | | | | 1 | | | | |
| Ernberg et al. (2011) | | | | | | | | | 1 | | | | |
| Gomes et al. (2014) | | | | | | | | 1 | | | | | |

*Supplementary Material S5.* Matrix of evidence with included studies per included systematic review. (Continued)

| *MATRIX OF EVIDENCE* | | | | | | | | | | | | | |
|---|---|---|---|---|---|---|---|---|---|---|---|---|---|
| *Primary Studies* | *Systematic Reviews* | | | | | | | | | | | | |
| *Study ID* | *Ågren et al.(2020)* | *Chen et al. (2023)* | *Cheng et al. (2022)* | *De la Torre Canales et al. (2017)* | *Fernández -Núñez et al. (2019)* | *Long et al. (2012)* | *Manfredini et al. (2015)* | *Miron et al. (2022)* | *Patel et al. (2019)* | *Rajamoorthy et al. (2023)* | *Sendra et al. (2021) & Sendra et al. (2022)* | *Sposito et al. (2014)* | *Zhang et al. (2024)* |
| Gomes et al. (2015) | | | | | | | | 1 | | | | | |
| Gouw et al. (2018) | | | | | | | | 1 | | | | | |
| Guarda-Nardini et al. (2008) | | 1 | 1 | 1 | 1 | 1 | | | 1 | 1 | 1 | 1 | 1 |
| Guarda-Nardini et al. (2012) | | | | | | | | | 1 | | | | |
| Hosgor et al. (2020) | | 1 | | | | | | | | | | | 1 |
| Jadhao et al. (2017) | 1 | 1 | 1 | | | | | | | 1 | 1 | | 1 |
| Kaya et al. (2021) | | 1 | | | | | | | | 1 | 1 | | 1 |
| Kef et al. (2021) | | 1 | | | | | | | | | | | 1 |
| Kurtoglu et al. (2008) | 1 | | | | | | | | 1 | | | | |
| Landry et al. (2009) | | | | | | | 1 | | | | | | |
| Lee et al. (2010) | 1 | | 1 | 1 | 1 | 1 | 1 | | 1 | | 1 | 1 | |
| Madani et al. (2013) | | | | | | | 1 | | | | | | |
| Mainieri et al. (2014) | | | | | | | 1 | | | | | | |
| Matsumoto et al. (2015) | | | | | | | 1 | | | | | | |

| | | | | | | | | | | | | | |
|---|---|---|---|---|---|---|---|---|---|---|---|---|---|
| Nixdorf et al. (2002) | | | | | | | | | 1 | | | | |

*Supplementary Material S5.* Matrix of evidence with included studies per included systematic review. (Continued)

| *MATRIX OF EVIDENCE* | | | | | | | | | | | | | |
|---|---|---|---|---|---|---|---|---|---|---|---|---|---|
| *Primary Studies* | *Systematic Reviews* | | | | | | | | | | | | |
| *Study ID* | *Ågren et al.(2020)* | *Chen et al. (2023)* | *Cheng et al. (2022)* | *De la Torre Canales et al. (2017)* | *Fernández-Núñez et al. (2019)* | *Long et al. (2012)* | *Manfredini et al. (2015)* | *Miron et al. (2022)* | *Patel et al. (2019)* | *Rajamoorthy et al. (2023)* | *Sendra et al. (2021) & Sendra et al. (2022)* | *Sposito et al. (2014)* | *Zhang et al. (2024)* |
| Ohmure et al. (2016) | | | | | | | | 1 | | | | | |
| Ondo et al. (2018) | 1 | | 1 | | | | | 1 | | 1 | 1 | | 1 |
| Patel et al. (2017) | | | | | | | | | 1 | | | | |
| Redaelli et al. (2011) | | | | 1 | | | | | | | | | |
| Saletu et al. (2010) | | | | | | | 1 | | | | | | |
| Sato et al. (2015) | | | | | | | 1 | | | | | | |
| Shim et al. (2014) | | | 1 | 1 | | | 1 | | | | 1 | | |
| Shim et al. (2020) | | | | | | | | 1 | | | 1 | | |
| Singh et al. (2015) | | | | | | | | 1 | | | | | |
| Sener et al. (2007) | | | | | | 1 | | | | | | | |
| Silva et al. (2022) | | | | | | | | | | | | | 1 |
| Sumiya et al. (2014) | | | | | | | 1 | | | | | | |
| Takahashi et al. (2013) | | | | | | | 1 | | | | | | |

| | | | | | | | | | | | | | |
|---|---|---|---|---|---|---|---|---|---|---|---|---|---|
| Valiente et al. (2015) | | | | | | | 1 | | | | | | |
| Von Lindern et al. (2003) | | | | | | | | | 1 | | | | |

*Supplementary Material S5.* Matrix of evidence with included studies per included systematic review. (Continued)

| *MATRIX OF EVIDENCE* | | | | | | | | | | | | | |
|---|---|---|---|---|---|---|---|---|---|---|---|---|---|
| *Primary Studies* | *Systematic Reviews* | | | | | | | | | | | | |
| *Study ID* | *Ågren et al.(2020)* | *Chen et al. (2023)* | *Cheng et al. (2022)* | *De la Torre Canales et al. (2017)* | *Fernández-Núñez et al. (2019)* | *Long et al. (2012)* | *Manfredini et al. (2015)* | *Miron et al. (2022)* | *Patel et al. (2019)* | *Rajamoorthy et al. (2023)* | *Sendra et al. (2021) & Sendra et al. (2022)* | *Sposito et al. (2014)* | *Zhang et al. (2024)* |
| Yurttutan et al. (2019) | | 1 | | | | | | | | | 1 | | 1 |
| Zhang et al. (2016) | | 1 | | | 1 | | | | 1 | | | | |

*Supplementary Material S6.* Checklist for the Preferred Reporting Items for Overviews of Reviews (PRIOR).

| *Topic* | *Item* |
|---|---|
| TITLE | |
| Title | 1. Identify the report as an overview of reviews. |
| *ABSTRACT* | |
| Abstract | 2. Provide a comprehensive and accurate summary of the purpose, methods, and results of the overview of reviews. |
| *INTRODUCTION* | |
| Rationale | 3. Describe the rationale for conducting the overview of reviews in the context of existing knowledge. |
| Objectives | 4. Provide an explicit statement of the objective(s) or question(s) addressed by the overview of reviews. |
| *METHODS* | |
| Eligibility criteria | 5a. Specify the inclusion and exclusion criteria for the overview of reviews. If supplemental primary studies were included, this should be stated, with a rationale. |
| | 5b. Specify the definition of 'systematic review' as used in the inclusion criteria for the overview of reviews. |
| Information sources | 6. Specify all databases, registers, websites, organizations, reference lists, and other sources searched or consulted to identify systematic reviews and supplemental primary studies (if included). Specify the date when each source was last searched or consulted. |
| Search strategy | 7. Present the full search strategies for all databases, registers and websites, such that they could be reproduced. Describe any search filters and limits applied. |
| Selection process | 8a. Describe the methods used to decide whether a systematic review or supplemental primary study (if included) met the inclusion criteria of the overview of reviews. |
| | 8b. Describe how overlap in the populations, interventions, comparators, and/or outcomes of systematic reviews was identified and managed during study selection. |
| Data collection process | 9a. Describe the methods used to collect data from reports. |
| | 9b. If applicable, describe the methods used to identify and manage primary study overlap at the level of the comparison and outcome during data collection. For each outcome, specify the method used to illustrate and/or quantify the degree of primary study overlap across systematic reviews. |
| | 9c. If applicable, specify the methods used to manage discrepant data across systematic reviews during data collection. |
| Data items | 10. List and define all variables and outcomes for which data were sought. Describe any assumptions made and/or measures taken to identify and clarify missing or unclear information. |

*Supplementary Material S6.* Checklist for the Preferred Reporting Items for Overviews of Reviews (PRIOR). (Continued)

| *Topic* | *Item* |
|---|---|
| Risk of bias assessment | 11a. Describe the methods used to assess risk of bias or methodological quality of the included systematic reviews. |
| | 11b. Describe the methods used to collect data on (from the systematic reviews) and/or assess the risk of bias of the primary studies included in the systematic reviews. Provide a justification for instances where flawed, incomplete, or missing assessments are identified but not re-assessed. |
| | 11c. Describe the methods used to assess the risk of bias of supplemental primary studies (if included). |
| Synthesis methods | 12a. Describe the methods used to summarize or synthesize results and provide a rationale for the choice(s). |
| | 12b. Describe any methods used to explore possible causes of heterogeneity among results. |
| | 12c. Describe any sensitivity analyses conducted to assess the robustness of the synthesized results. |
| Reporting bias assessment | 13. Describe the methods used to collect data on (from the systematic reviews) and/or assess the risk of bias due to missing results in a summary or synthesis (arising from reporting biases at the levels of the systematic reviews, primary studies, and supplemental primary studies, if included). |
| Certainty assessment | 14. Describe the methods used to collect data on (from the systematic reviews) and/or assess certainty (or confidence) in the body of evidence for an outcome. |
| *RESULTS* | |
| Systematic review and supplemental primary study selection | 15a. Describe the results of the search and selection process, including the number of records screened, assessed for eligibility, and included in the overview of reviews, ideally with a flow diagram. |
| | 15b. Provide a list of studies that might appear to meet the inclusion criteria, but were excluded, with the main reason for exclusion. |
| Characteristics of systematic reviews and supplemental primary studies | 16. Cite each included systematic review and supplemental primary study (if included) and present its characteristics. |
| Primary study overlap | 17. Describe the extent of primary study overlap across the included systematic reviews. |
| Risk of bias in systematic reviews, primary studies, and supplemental primary studies | 18a. Present assessments of risk of bias or methodological quality for each included systematic review. |
| | 18b. Present assessments (collected from systematic reviews or assessed anew) of the risk of bias of the primary studies included in the systematic reviews. |
| | 18c. Present assessments of the risk of bias of supplemental primary studies (if included). |

*Supplementary Material S6.* Checklist for the Preferred Reporting Items for Overviews of Reviews (PRIOR). (Continued)

| *Topic* | *Item* |
|---|---|
| Summary or synthesis of results | 19a. For all outcomes, summarize the evidence from the systematic reviews and supplemental primary studies (if included). If meta-analyses were done, present for each the summary estimate and its precision and measures of statistical heterogeneity. If comparing groups, describe the direction of the effect.<br>19b. If meta-analyses were done, present results of all investigations of possible causes of heterogeneity.<br>19c. If meta-analyses were done, present results of all sensitivity analyses conducted to assess the robustness of synthesized results. |
| Reporting biases | 20. Present assessments (collected from systematic reviews and/or assessed anew) of the risk of bias due to missing primary studies, analyses, or results in a summary or synthesis (arising from reporting biases at the levels of the systematic reviews, primary studies, and supplemental primary studies, if included) for each summary or synthesis assessed. |
| Certainty of evidence | 21. Present assessments (collected or assessed anew) of certainty (or confidence) in the body of evidence for each outcome. |
| *DISCUSSION* | |
| Discussion | 22a. Summarize the main findings, including any discrepancies in findings across the included systematic reviews and supplemental primary studies (if included).<br>22b. Provide a general interpretation of the results in the context of other evidence.<br>22c. Discuss any limitations of the evidence from systematic reviews, their primary studies, and supplemental primary studies (if included) included in the overview of reviews. Discuss any limitations of the overview of reviews methods used.<br>22d. Discuss implications for practice, policy, and future research (both systematic reviews and primary research). Consider the relevance of the findings to the end users of the overview of reviews, e.g., healthcare providers, policymakers, patients, among others. |
| *OTHER INFORMATION* | |
| Registration and protocol | 23a. Provide registration information for the overview of reviews, including register name and registration number, or state that the overview of reviews was not registered.<br>23b. Indicate where the overview of reviews protocol can be accessed, or state that a protocol was not prepared.<br>23c. Describe and explain any amendments to information provided at registration or in the protocol. Indicate the stage of the overview of reviews at which amendments were made. |
| Support | 24. Describe sources of financial or non-financial support for the overview of reviews, and the role of the funders or sponsors in the overview of reviews. |
| Competing interests | 25. Declare any competing interests of the overview of reviews' authors. |

*Supplementary Material S6.* Checklist for the Preferred Reporting Items for Overviews of Reviews (PRIOR). (Continued)

| *Topic* | *Item* |
|---|---|
| Author information | 26a. Provide contact information for the corresponding author. |
| | 26b. Describe the contributions of individual authors and identify the guarantor of the overview of reviews. |
| Availability of data and other materials | 27. Report which of the following are available, where they can be found, and under which conditions they may be accessed: template data collection forms; data collected from included systematic reviews and supplemental primary studies; analytic code; any other materials used in the overview of reviews. |

*Supplementary Material S7.* Search strategies.

| *DATABASE* | *QUERY* |
|---|---|
| *Cochrane Library* | ("Botulinum A Toxin" OR "Botulinum Toxin" OR "Botulinum Neurotoxin A" OR "Botox" OR "Botulinum A Toxin" OR "Botulinum Toxins" OR "Botulinum Neurotoxins" OR "Botulinum Neurotoxin-A" OR "Botulinum Neurotoxin" OR "Botulin" OR "Onabotulinum Toxin" OR "Botulinum Injections" OR "Botulinum Injection" OR "Botulinum-A" OR "BoNTA" OR "BTX" OR "BoNT-A" OR "BoNT" OR "praBTX-A" OR "BT-A" OR "BTX-A" OR "BoNT-A"):ti,ab,kw AND ("Meta-Analysis" OR "Meta Analysis" OR "Metanalysis" OR "Review"):ti,ab,kw AND (Bruxism):ti,ab,kw |
| *EMBASE* | ('Botulinum Toxin'/exp OR 'Botulinum Toxin' OR 'Botulinum Neurotoxin a'/exp OR 'Botulinum Neurotoxin A' OR 'Botox'/exp OR 'Botox' OR 'Botulinum A Toxin'/exp OR 'Botulinum A Toxin' OR 'Botulinum Toxins'/exp OR 'Botulinum Toxins' OR 'Botulinum Neurotoxins' OR 'Botulinum Neurotoxin-A'/exp OR 'Botulinum Neurotoxin-A' OR 'Botulinum Neurotoxin'/exp OR 'Botulinum Neurotoxin' OR 'Botulin'/exp OR 'Botulin' OR 'Onabotulinum Toxin' OR 'Botulinum Injections' OR 'Botulinum Injection' OR 'Botulinum-A' OR 'BoNTA' OR 'BTX'/exp OR 'BTX' OR 'BoNT' OR 'praBTX-A' OR 'BT-A' OR 'BTX-A' OR 'BoNT-A'/exp OR 'BoNT-A') AND ('Bruxism'/exp OR Bruxism) AND ('Meta-Analysis'/exp OR 'Meta-Analysis' OR 'Meta Analysis'/exp OR 'Meta Analysis' OR 'Metanalysis' OR 'Review'/exp OR 'Review') |
| *LILACS* | ("Botulinum A Toxin" OR "Botulinum Toxin" OR "Botulinum Neurotoxin A" OR "Botox" OR "Botulinum A Toxin" OR "Botulinum Toxins" OR "Botulinum Neurotoxins" OR "Botulinum Neurotoxin-A" OR "Botulinum Neurotoxin" OR "Botulin" OR "Onabotulinum Toxin" OR "Botulinum Injections" OR "Botulinum Injection" OR "Botulinum-A" OR "BoNTA" OR "BTX" OR "BoNT-A" OR "BoNT" OR "praBTX-A" OR "BT-A" OR "BTX-A" OR "BoNT-A" OR "Toxinas Botulínicas Tipo A" OR "Neurotoxina Botulínica Tipo A" OR "Onabotulinumtoxina A" OR "OnabotulinumtoxinaA" OR "Toxina Botulínica A" OR "Toxina Botulínica Tipo A" OR "Toxinas Botulínicas" OR "Neurotoxina Botulínica" OR "Neurotoxinas Botulínicas") AND ("Bruxism" OR "Bruxismo") AND ("Meta-Analysis" OR "Meta Analysis" OR "Metanalise" OR "Meta-Análise" OR "Metanalysis" OR "Review" OR "Revisão" OR "Revisión") AND (DB:("LILACS")) |
| *Livivo* | ("Botulinum A Toxin" OR "Botulinum Toxin" OR "Botulinum Neurotoxin A" OR "Botox" OR "Botulinum A Toxin" OR "Botulinum Toxins" OR "Botulinum Neurotoxins" OR "Botulinum Neurotoxin-A" OR "Botulinum Neurotoxin" OR "Botulin" OR "Onabotulinum Toxin" OR "Botulinum Injections" OR "Botulinum Injection" OR "Botulinum-A" OR "BoNTA" OR "BTX" OR "BoNT-A" OR "BoNT" OR "praBTX-A" OR "BT-A" OR "BTX-A" OR "BoNT-A") AND (Bruxism) AND ("Meta-Analysis" OR "Meta Analysis" OR "Metanalysis" OR "Review") |

*Supplementary Material S7.* Search strategies. (Continued)

| | |
|---|---|
| *PubMed via MEDLINE* | (("Botulinum Toxins, Type A"[MeSH Terms] OR "Botulinum Toxins"[MeSH Terms] OR "Botulinum Toxins" OR "Botox" OR "Botulinum A Toxin" OR "Botulinum Neurotoxins" OR "Botulinum Toxin" OR "Botulinum neurotoxin-A" OR "Botulinum Neurotoxin" OR "Botulin" OR "Onabotulinum toxin" OR "Botulinum injections" OR "Botulinum injection" OR "botulinum-A" OR "BONTA" OR "BTX" OR "BoNT-A" OR "BoNT" OR "praBTX-A" OR "BT-A" OR "BTX-A" OR "BONT-A") AND ("Bruxism"[MeSH Terms] OR "Bruxism") AND ("Meta-Analysis"[Title/Abstract] OR "Meta Analysis"[Title/Abstract] OR "MetaAnalysis"[Title/Abstract] OR "Review"[Title/Abstract])) |
| *Scopus* | TITLE-ABS-KEY("Botulinum A Toxin" OR "Botulinum Toxin" OR "Botulinum Neurotoxin A" OR "Botox" OR "Botulinum A Toxin" OR "Botulinum Toxins" OR "Botulinum Neurotoxins" OR "Botulinum Neurotoxin-A" OR "Botulinum Neurotoxin" OR "Botulin" OR "Onabotulinum Toxin" OR "Botulinum Injections" OR "Botulinum Injection" OR "Botulinum-A" OR "BoNTA" OR "BTX" OR "BoNT-A" OR "BoNT" OR "praBTX-A" OR "BT-A" OR "BTX-A" OR "BoNT-A") AND TITLE-ABS-KEY (Bruxism) AND TITLE-ABS-KEY ("Meta-Analysis" OR "Meta Analysis" OR "Metanalysis" OR "Review") |
| *Web of Science* | TS=("Botulinum A Toxin" OR "Botulinum Toxin" OR "Botulinum Neurotoxin A" OR "Botox" OR "Botulinum A Toxin" OR "Botulinum Toxins" OR "Botulinum Neurotoxins" OR "Botulinum Neurotoxin-A" OR "Botulinum Neurotoxin" OR "Botulin" OR "Onabotulinum Toxin" OR "Botulinum Injections" OR "Botulinum Injection" OR "Botulinum-A" OR "BoNTA" OR "BTX" OR "BoNT-A" OR "BoNT" OR "praBTX-A" OR "BT-A" OR "BTX-A" OR "BoNT-A") AND TS=(Bruxism) AND TS=("Meta-Analysis" OR "Meta Analysis" OR "Metanalysis" OR "Review") |
| *Google Scholar* | (Bruxism) AND ("Botulinum Toxins Type A") AND ("Meta-Analysis" OR "Meta Analysis" OR "Metanalysis" OR "Review") |
| *ProQuest D&T Global* | noft("Botulinum A Toxin" OR "Botulinum Toxin" OR "Botulinum Neurotoxin A" OR "Botox" OR "Botulinum A Toxin" OR "Botulinum Toxins" OR "Botulinum Neurotoxins" OR "Botulinum Neurotoxin-A" OR "Botulinum Neurotoxin" OR "Botulin" OR "Onabotulinum Toxin" OR "Botulinum Injections" OR "Botulinum Injection" OR "Botulinum-A" OR "BoNTA" OR "BTX" OR "BoNT-A" OR "BoNT" OR "praBTX-A" OR "BT-A" OR "BTX-A" OR "BoNT-A") AND noft(Bruxism) AND noft("Meta-Analysis" OR "Meta Analysis" OR "Metanalysis" OR "Review") |

Source: Authors, 2023.

*Supplementary Material S8.* Graphical Representation of Overlap for OVErviews (GROOVE) Tool.

**MATRIX OF EVIDENCE**

**TOPIC:**

| Primary Studies | | Systematic Reviews | | | | | | | | | | | | |
|---|---|---|---|---|---|---|---|---|---|---|---|---|---|---|
| Study ID | Reference | Agren et al.(202 | Chen et al. (202 | Cheng et al. (20 | De la Torre Can | Fernandez-Nuñ | Long et al. (201 | Manfredini et al | Miron et al. (202 | Patel et al. (2019 | Rajamoorthy et | Sendra et al. (20 | Sposito et al. (2 | Zhang et al. (20 |
| Abekura et al. (2006) | | | | | | | 1 | | | | | | | |
| Ali et al. (2021) | | | | | | | | | | | 1 | | | |
| Al-Wayli et al. (2017) | | 1 | 1 | | 1 | | | | | 1 | 1 | | 1 | 1 |
| Al-Wayli et al. (2024) | | 1 | | | | | | | | | 1 | | 1 | 1 |
| Arima et al. (2012) | | | | | | | 1 | | | | | | | |
| Asutay et al. (2017) | | 1 | | | | | | | | | | | 1 | 1 |
| Baughman et al. (2014) | | | | | | | | | | 1 | | | | |
| Bergmann et al. (2020) | | | | | | | | 1 | | | | | | |
| Bolayir et al. (2005) | | | | 1 | | 1 | | | | | | | | |
| Carli et al. (2016) | | | | | | | | | 1 | | | | | |
| Carra et al. (2010) | | | | | | | 1 | | | | | | | |
| Cahlin et al. (2016) | | | | | | | | 1 | | | | | | |
| Chaurand et al. (2017) | | | | | | | | | 1 | | | | | |
| Ernberg et al. (2011) | | | | | | | | | 1 | | | | | |
| Gomes et al. (2014) | | | | | | | | 1 | | | | | | |
| Gomes et al. (2015) | | | | | | | | 1 | | | | | | |
| Gouw et al. (2016) | | | | | | | | 1 | | | | | | |
| Guarda-Nardini et al. (2006) | | 1 | 1 | 1 | 1 | 1 | | | 1 | 1 | 1 | 1 | 1 | 1 |
| Guarda-Nardini et al. (2012) | | | | | | | | | 1 | | | | | |
| Hosgor et al. (2020) | | 1 | | | | | | | | | | | 1 | 1 |
| Jadhao et al. | 1 | 1 | 1 | | | | | | | 1 | 1 | | 1 | 1 |
| Kaya et al. (2021) | | 1 | | | | | | | | 1 | 1 | | 1 | 1 |
| Kef et al. (2021) | | 1 | | | | | | | | | | | 1 | 1 |
| Kurtoglu et a | 1 | | | | | | | | 1 | | | | | |
| Landry et al. (2009) | | | | | | | 1 | | | | | | | |
| Lee et al. (20 | 1 | | 1 | 1 | 1 | 1 | 1 | | 1 | | 1 | 1 | | |
| Madani et al. (2013) | | | | | | | 1 | | | | | | | |
| Mainieri et al. (2014) | | | | | | | 1 | | | | | | | |
| Matsumoto et al. (2015) | | | | | | | 1 | | | | | | | |
| Nixdorf et al. (2002) | | | | | | | | | 1 | | | | | |
| Ohmure et al. (2016) | | | | | | | | 1 | | | | | | |
| Ondo et al. ( | 1 | | 1 | | | | | 1 | | 1 | 1 | | 1 | 1 |
| Patel et al. (2017) | | | | | | | | | 1 | | | | | |
| Redaelli et al. (2011) | | | | 1 | | | | | | | | | | |
| Saletu et al. (2010) | | | | | | | 1 | | | | | | | |
| Sato et al. (2015) | | | | | | | 1 | | | | | | | |
| Shim et al. (2014) | | | 1 | 1 | | | 1 | | | | 1 | | | |
| Shim et al. (2020) | | | | | | | | 1 | | | 1 | | | |
| Singh et al. (2015) | | | | | | | | 1 | | | | | | |
| Sener et al. (2007) | | | | | | 1 | | | | | | | | |
| Silva et al. (2022) | | | | | | | | | | | | | 1 | 1 |
| Sumiya et al. (2014) | | | | | | | 1 | | | | | | | |
| Takahashi et al. (2013) | | | | | | | 1 | | | | | | | |
| Valiente et al. (2015) | | | | | | | 1 | | | | | | | |
| Von Lindern et al. (2003) | | | | | | | | | 1 | | | | | |
| Yurttutan et al. (2019) | | 1 | | | | | | | | | 1 | | 1 | 1 |
| Zhang et al. (2016) | | 1 | | | 1 | | | | 1 | | | | | |

**A**

**Overall results**

| | | |
|---|---|---|
| Number of columns (number of reviews) | c | 13 |
| Number of rows (number of index publications) | r | 47 |
| er of included primary studies (including double counting) | N | 104 |
| Covered area | N/(rc) | 17,02% |
| Corrected covered area | (N-r)/(rc-r) | 10,11% |
| Interpretation of overlap | High overlap | |
| Structural Zeros | X | 0 |
| Corrected covered area (adjusting by structural zeros) | (N-r)/(rc-r-X) | 10,11% |
| N° of non-overlapped primary studies | In 1 SR | 31 |
| Number of overlapped primary studies | In 2 SRs | 3 |
| | In 3 SRs | 4 |
| | In 4 SRs | 3 |
| | In 5 SRs | 1 |
| | In 6 SRs | 2 |
| | In 7 SRs | 1 |
| | In 8 SRs | 1 |
| | In 9 SRs | 0 |
| | In 10 SRs | 0 |
| | In 11 SRs | 1 |
| | In 12 SRs | 0 |
| | In 13 SRs | 0 |
| | In 14 SRs | 0 |
| | n 15 or more SR | 0 |

**Graphical Representation of Overlap for OVErviews (GROOVE)**

| | Agren et al. (202 | Chen et al. (202 | Cheng et al. (2022) | De la Torre Canales et al. ( | Fernández-Núñez et al. (2019) | Long et al. (2012) | Manfredini et al. (2015) | Miron et al. (2022) | Patel et al. (2019) | Rajamoorthy et al. (2023) | Sendra et al. (2021) & Sendra et | Sposito et al. (2014) |
|---|---|---|---|---|---|---|---|---|---|---|---|---|
| Chen et al. (2023) | 23,1% | | | | | | | | | | | |
| Cheng et al. (2022) | 7,1% | 37,5% | | | | | | | | | | |
| De la Torre Canales et al. (2017) | 27,3% | 42,9% | 28,6% | | | | | | | | | |
| Fernández-Núñez et al. (2019) | 7,7% | 25,0% | 50,0% | 33,3% | | | | | | | | |
| Long et al. (2012) | 0,0% | 11,1% | 11,8% | 5,9% | 5,9% | | | | | | | |
| Manfredini et al. (2015) | 0,0% | 7,1% | 0,0% | 0,0% | 0,0% | 0,0% | | | | | | |
| Miron et al. (2022) | 10,5% | 13,3% | 14,3% | 25,0% | 15,4% | 4,2% | 0,0% | | | | | |
| Patel et al. (2019) | 33,3% | 50,0% | 10,0% | 25,0% | 11,1% | 0,0% | 7,1% | 6,3% | | | | |
| Rajamoorthy et al. (2023) | 40,0% | 54,5% | 23,1% | 25,0% | 15,4% | 8,7% | 11,1% | 10,0% | 41,7% | | | |
| Sendra et al. (2021) & Sendra et al. (2022) | 9,1% | 33,3% | 40,0% | 50,0% | 50,0% | 6,7% | 0,0% | 18,2% | 14,3% | 18,2% | | |
| Sposito et al. (2014) | 75,0% | 30,8% | 6,7% | 15,4% | 7,1% | 0,0% | 5,3% | 4,8% | 41,7% | 46,7% | 8,3% | |
| Zhang et al. (2024) | 75,0% | 30,8% | 6,7% | 15,4% | 7,1% | 0,0% | 5,3% | 4,8% | 41,7% | 46,7% | 8,3% | 100,0% |

| | | |
|---|---|---|
| 78 | = | Total nodes (pairs of revie |
| 14 | = | Slight overlap (<5%) |
| 18 | = | Moderate overlap (5% to <10%) |
| 10 | = | High overlap (10% to <15%) |
| 36 | = | Very High overlap (≥15%) |

**B**

## Overall results

| | | |
|---|---|---|
| Number of columns (number of reviews) | c | 13 |
| Number of rows (number of index publications) | r | 47 |
| ber of included primary studies (including double counting) | N | 104 |
| Covered area | N/(rc) | 17,02% |
| Corrected covered area | (N-r)/(rc-r) | 10,11% |
| Interpretation of overlap | High overlap | |
| Structural Zeros | X | 0 |
| Corrected covered area (adjusting by structural zeros) | (N-r)/(rc-r-X) | 10,11% |
| N° of non-overlapped primary studies | In 1 SR | 31 |
| Number of overlapped primary studies | In 2 SRs | 3 |
| | In 3 SRs | 4 |
| | In 4 SRs | 3 |
| | In 5 SRs | 1 |
| | In 6 SRs | 2 |
| | In 7 SRs | 1 |
| | In 8 SRs | 1 |
| | In 9 SRs | 0 |
| | In 10 SRs | 0 |
| | In 11 SRs | 1 |
| | In 12 SRs | 0 |
| | In 13 SRs | 0 |
| | In 14 SRs | 0 |
| | n 15 or more SR: | 0 |

## Graphical Representation of Overlap for OVErviews (GROOVE)

| | Agren et al. (202 | Chen et al. (202 | Cheng et al. (2022) | De la Torre Canales et al. ( | Fernández-Núñez et al. (2019) | Long et al. (2012) | Manfredini et al. (2015) | Miron et al. (2022) | Patel et al. (2019) | Rajamoorthy et al. (2023) | Sendra et al. (2021) & Sendra et | Sposito et al. (2014) |
|---|---|---|---|---|---|---|---|---|---|---|---|---|
| Chen et al. (2023) | 23,1% | | | | | | | | | | | |
| Cheng et al. (2022) | 7,1% | 37,5% | | | | | | | | | | |
| De la Torre Canales et al. (2017) | 27,3% | 42,9% | 28,6% | | | | | | | | | |
| Fernández-Núñez et al. (2019) | 7,7% | 25,0% | 50,0% | 33,3% | | | | | | | | |
| Long et al. (2012) | 0,0% | 11,1% | 11,8% | 5,9% | 5,9% | | | | | | | |
| Manfredini et al. (2015) | 0,0% | 7,1% | 0,0% | 0,0% | 0,0% | 0,0% | | | | | | |
| Miron et al. (2022) | 10,5% | 13,3% | 14,3% | 25,0% | 15,4% | 4,2% | 0,0% | | | | | |
| Patel et al. (2019) | 33,3% | 50,0% | 10,0% | 25,0% | 11,1% | 0,0% | 7,1% | 6,3% | | | | |
| Rajamoorthy et al. (2023) | 40,0% | 54,5% | 23,1% | 25,0% | 15,4% | 8,7% | 11,1% | 10,0% | 41,7% | | | |
| Sendra et al. (2021) & Sendra et al. (2022) | 9,1% | 33,3% | 40,0% | 50,0% | 50,0% | 6,7% | 0,0% | 18,2% | 14,3% | 18,2% | | |
| Sposito et al. (2014) | 75,0% | 30,8% | 6,7% | 15,4% | 7,1% | 0,0% | 5,3% | 4,8% | 41,7% | 46,7% | 8,3% | |
| Zhang et al. (2024) | 75,0% | 30,8% | 6,7% | 15,4% | 7,1% | 0,0% | 5,3% | 4,8% | 41,7% | 46,7% | 8,3% | 100,0% |

| | | |
|---|---|---|
| 78 | = | **Total nodes (pairs of review** |
| 14 | = | Slight overlap (<5%) |
| 18 | = | Moderate overlap (5% to <10%) |
| 10 | = | High overlap (10% to <15%) |
| 36 | = | Very High overlap (≥15%) |

C

A: Tab 1 of the Microsoft Excel tool displaying the initial GROOVE assessment parameters.

B: Tab 2 of the Microsoft Excel tool showing detailed calculations and data input for GROOVE analysis (colored version).

C: Tab 3 of the Microsoft Excel tool showing detailed calculations and data input for GROOVE analysis (greyscale version).

Source: Authors, 2024.

References:

1. Anandan C, Jankovic J. Botulinum Toxin in Movement Disorders: An Update. *Toxins* **2021**, 13(1), 42-73.
2. Balanta-Melo J, Vargas JP, Bendersky J, Villanueva J. Toxina Botulínica tipo A para el bruxismo del sueño en adultos. *Int. J. Interdiscip. Dent.* **2022**, 15(1), 101-107.
3. Bardavid ECL. Revisión sistemática: estudio comparativo entre férulas y botox como tratamiento en pacientes bruxistas. "URL: https://titula.universidadeuropea.com/handle/20.500.12880/1660?show=full (Acessed on 16 June 2024)".
4. Buzatu R, Luca MM, Castiglione L, Sinescu C. Efficacy and Safety of Botulinum Toxin in the Management of Temporomandibular Symptoms Associated with Sleep Bruxism: A Systematic Review. *Dent. J.* **2024**, 12(6), 156-169.
5. Cunha FR, Borba DBM, Oliveira RCG, Oliveira RC, Valarelli FP, Freitas KMS, Cotrin P. The use of botulinum toxin in the treatment of bruxism. *Res., Soc. Dev.* **2022**, 11(4), e34011427304.
6. da Silva Ramalho JA, Palma LF, Ramalho KM, Tedesco TK, Morimoto S. Effect of botulinum toxin A on pain, bite force, and satisfaction of patients with bruxism: A randomized single-blind clinical trial comparing two protocols. *Saudi Dent. J.* **2023**, 35(1), 53-60.
7. De Lima LR, Nechita LHP, Paulista B. Toxina botulínica na odontologia: tratamento de bruxismo. Revisão de literatura. "URL: https://lyceumonline.usf.edu.br/salavirtual/documentos/3453.pdf (Acessed on 16 June 2024)".
8. Dimartini S, Algayer E, Mioso F, Thereza-Bussolaro C. Toxin botulinum for therapeutic management of bruxism. *Craniofac. Res. Connect. J.* **2022**, 2(1), 72-80.
9. Ferreira LC, de Almeida Coelho J. O uso terapêutico da toxina botulínica para tratamentos de bruxismo. *Rev. Cient. Unilago* **2023**, 1(1), 1-9.
10. Khawja ST. A systematic review: the use of botulinum toxin A for the treatment of masseter hypertrophy and masticatory myofascial pain associated with bruxism. "URL: https://open.bu.edu/bitstream/handle/2144/48703/Khawja_bu_0017N_18834.pdf?sequence=6&isAllowed=y (Acessed on 16 June 2024)".
11. Kumar A, Spivakovsky S. Bruxism- is botulinum toxin an effective treatment? *Evid. Based Dent.* **2018**, 19(2), 59-59.
12. Macedo CR, Silva AB, Machado MA, Saconato H, Prado GF. Occlusal splints for treating sleep bruxism (tooth grinding). *Cochrane Database Syst. Rev.* **2007**, 2007(4), CD005514.
13. Minakuchi H, Fujisawa M, Abe Y, et al. Managements of sleep bruxism in adult: A systematic review. *Jpn. Dent. Sci. Rev.* **2022**, 58, 124-136.
14. Sierra Betancourth C, Ramos Cruz MC, Castrillón Arango JT. Revisión narrativa. Eficacia de una placa neuromiorelajante en comparación con la toxina botulínica para pacientes con bruxismo. "URL: https://repositorio.uan.edu.co/server/api/core/bitstreams/17701f09-411b-4e1c-b77f-5e71784cc1b1/content ((Acessed on 16 June 2024)".
15. Teixeira SAF, de Mello Sposito MM. A utilização de Toxina Onabotulínica A para bruxismo: revisão de literatura. *Rev. Bras. Odontol.* **2014**, 70(2), 202-204.
16. Tinastepe N, Küçük BB, Oral K. Botulinum toxin for the treatment of bruxism. *Cranio.* **2015**, 33(4), 292-299.
17. Ågren, M.; Sahin, C.; Pettersson, M. The Effect of Botulinum Toxin Injections on Bruxism: A Systematic Review. *J. Oral Rehabil.* **2020**, *47*, 395–402.
18. Chen, Y.; Tsai, C.-H.; Bae, T.H.; Huang, C.-Y.; Chen, C.; Kang, Y.-N.; Chiu, W.-K. Effectiveness of Botulinum Toxin Injection on Bruxism: A SystematicReview and Meta-Analysis of Randomized Controlled Trials. *Aesthetic Plast. Surg.* **2023**, *47*, 775–790.
19. Cheng, Y.; Yuan, L.; Ma, L.; Pang, F.; Qu, X.; Zhang, A. Efficacy of Botulinum-A for Nocturnal Bruxism Pain and the Occurrence of Bruxism Events: AMeta-Analysis and Systematic Review. *Br. J. Oral Maxillofac. Surg.* **2022**, *60*, 174–182.
20. De la Torre Canales, G.; Câmara-Souza, M.B.; Do Amaral, C.F.; Garcia, R.C.M.R.; Manfredini, D. Is There Enough Evidence to Use Botulinum ToxinInjections for Bruxism Management? A Systematic Literature Review. *Clin. Oral Investig.* **2017**, *21*, 727–734.
21. Fernández-Núñez, T.; Amghar-Maach, S.; Gay-Escoda, C. Efficacy of Botulinum Toxin in the Treatment of Bruxism: Systematic Review. *Med. Oral Patol. Oral Y Cir. Bucal* **2019**, *24*, e416.
22. Long, H.; Liao, Z.; Wang, Y.; Liao, L.; Lai, W. Efficacy of Botulinum Toxins on Bruxism: An Evidence-based Review. *Int. Dent. J.* **2012**, *62*, 1–5.
23. Miron, M.I.; Ciora, E.; Vedinas, T.; Mocuta, D.-E. Therapeutic Approaches to Nocturnal Bruxism—A Systematic Review. *Arch. Balk. Med. Union* **2022**, *57*, 372–383.
24. Patel, J.; Cardoso, J.A.; Mehta, S. A Systematic Review of Botulinum Toxin in the Management of Patients with Temporomandibular Disorders andBruxism. *Br. Dent. J.* **2019**, *226*, 667–672.
25. Rajamoorthy, S.N. Botulinum Toxin-A Injections into Facial Muscles for the Treatment of Temporomandibular Disorders and Bruxism: A SystematicReview. *J. Popul. Ther. Clin. Pharmacol.* **2023**, *30*, 428–452.
26. Sendra, L.A.; Montez, C.; Vianna, K.C.; Barboza, E.P. Clinical Outcomes of Botulinum Toxin Type A Injections in the Management of Primary Bruxism inAdults: A Systematic Review. *J. Prosthet. Dent.* **2021**, *126*, 33–40.
27. Sendra, L.A.; Antunes, L.A.A.; Barboza, E.P. Use of Botulinum Neurotoxin Type A in the Management of Primary Bruxism in Adults: An UpdatedSystematic Review. *J. Prosthet. Dent.* **2024**, *132*, 93–99.

28. Sposito, M.M.d.M.; Teixeira, S.A.F. Botulinum Toxin A for Bruxism: A Systematic Review. *Acta Fisiátrica* **2014**, *21*, 201–204.
29. Zhang, A.; Zhang, J.; Zhou, X.; Sun, L.; Li, T. Can Botulinum Toxin Injection Alleviate the Pain of Bruxism? A Bayesian Network Analysis and a Single-Arm Analysis. *J. Dent. Sci.* **2024**, *19*, 885–893.
30. Abekura H, Yokomura M, Sadamori S, Hamada T. The initial effects of occlusal splint vertical thickness on the nocturnal EMG activities of masticatory muscles in subjects with a bruxism habit. *Int. J. Prosthodont.* **2008**, 21(2), 116-120.
31. Ali SM, Alqutaibi AY, Aboalrejal A, Elawady DM. Botulinum toxin and occlusal splints for the management of sleep bruxism in individuals with implant overdentures: A randomized controlled trial. *Saudi Dent. J.* **2021**, 33(8), 1004-1011.
32. Al-Wayli H. Treatment of chronic pain associated with nocturnal bruxism with botulinum toxin. A prospective and randomized clinical study. *J. Clin. Exp. Dent.* **2017**, 9(1), e112-e117.
33. Al-wayli H, Abdulrahman BI, Rastogi S. Does botulinum toxin have any role in the management of chronic pain associated with bruxism?. *Cranio*. **2024**, 42(2), 215-222.
34. Arima T, Tomonaga A, Toyota M, Inoue SI, Ohata N, Svensson P. Does restriction of mandibular movements during sleep influence jaw-muscle activity?. *J. Oral. Rehabil.* **2012,** 39(7), 545-551.
35. Asutay F, Atalay Y, Asutay H, Acar AH. The Evaluation of the Clinical Effects of Botulinum Toxin on Nocturnal Bruxism. *Pain Res. Manag.* **2017**, 2017, 6264146-6264150.
36. Baughman et al. (2014) - retracted.
37. Bergmann A, Edelhoff D, Schubert O, Erdelt KJ, Pho Duc JM. Effect of treatment with a full-occlusion biofeedback splint on sleep bruxism and TMD pain: a randomized controlled clinical trial. *Clin. Oral Investig.* **2020**, 24(11), 4005-4018.
38. Bolayir G, Bolayir E, Coskun A, Özdemir AK, Topaktas S. Botulinum toxin type-A practice in bruxism cases. *Neurol. Psychiatr. Brain Res.* **2005**, 12, 43-46.
39. De Carli BM, Magro AK, Souza-Silva BN, et al. The effect of laser and botulinum toxin in the treatment of myofascial pain and mouth opening: A randomized clinical trial. *J. Photochem. Photobiol B.* **2016**, 159, 120-123.
40. Carra MC, Macaluso GM, Rompré PH, et al. Clonidine has a paradoxical effect on cyclic arousal and sleep bruxism during NREM sleep. *Sleep*. **2010**, 33(12), 1711-1716.
41. Cahlin BJ, Hedner J, Dahlström L. A randomised, open-label, crossover study of the dopamine agonist, pramipexole, in patients with sleep bruxism. *J. Sleep Res.* **2017**, 26(1), 64-72.
42. Chaurand J, Pacheco-Ruíz L, Orozco-Saldívar H, López-Valdés J. Efficacy of botulinum toxin therapy in treatment of myofascial pain. *J. Appl. Oral Sci.* **2017**, 59(3), 351-356.
43. Ernberg M, Hedenberg-Magnusson B, List T, Svensson P. Efficacy of botulinum toxin type A for treatment of persistent myofascial TMD pain: a randomized, controlled, double-blind multicenter study. *Pain*. **2011**, 152(9), 1988-1996.
44. Gomes CA, El Hage Y, Amaral AP, Politti F, Biasotto-Gonzalez DA. Effects of massage therapy and occlusal splint therapy on electromyographic activity and the intensity of signs and symptoms in individuals with temporomandibular disorder and sleep bruxism: a randomized clinical trial. *Chiropr. Man. Therap.* **2014**, 22(1), 43-50.
45. Gomes CAFP, El-Hage Y, Amaral AP, et al. Effects of Massage Therapy and Occlusal Splint Usage on Quality of Life and Pain in Individuals with Sleep Bruxism: A Randomized Controlled Trial. *J. Jpn. Phys. Ther. Assoc.* **2015**, 18(1), 1-6.
46. Gouw S, de Wijer A, Kalaykova SI, Creugers NHJ. Masticatory muscle stretching for the management of sleep bruxism: A randomised controlled trial. *J. Oral Rehabil.* **2018**;45(10):770-776.
47. Guarda-Nardini L, Manfredini D, Salamone M, Salmaso L, Tonello S, Ferronato G. Efficacy of botulinum toxin in treating myofascial pain in bruxers: a controlled placebo pilot study. *Cranio*. **2008**, 26(2), 126-135.
48. Guarda-Nardini L, Stecco A, Stecco C, Masiero S, Manfredini D. Myofascial pain of the jaw muscles: comparison of short-term effectiveness of botulinum toxin injections and fascial manipulation technique. *Cranio*. **2012**, 30(2), 95-102.
49. Hosgor H, Altindis S. Efficacy of botulinum toxin in the management of temporomandibular myofascial pain and sleep bruxism. *J. Korean Assoc. Oral Maxillofac. Surg.* **2020**, 46(5), 335-340.
50. Jadhao VA, Lokhande N, Habbu SG, Sewane S, Dongare S, Goyal N. Efficacy of botulinum toxin in treating myofascial pain and occlusal force characteristics of masticatory muscles in bruxism. *Indian J. Dent. Res.* **2017**, 28(5), 493-497.
51. Kaya DI, Ataoglu H. Botulinum toxin treatment of temporomandibular joint pain in patients with bruxism: A prospective and randomized clinical study. *Niger. J. Clin. Pract.* **2021**, 24(3), 412-417.
52. Kef K. Application of Botulinum Toxin in Patients with Secondary Otalgia Caused by Bruxism. *J. Pain Res.* **2021**, 14, 1051-1059.
53. Kurtoglu C, Gur OH, Kurkcu M, Sertdemir Y, Guler-Uysal F, Uysal H. Effect of botulinum toxin-A in myofascial pain patients with or without functional disc displacement. *J. Oral Maxillofac. Surg.* **2008**, 66(8), 1644-1651.

54. Landry-Schönbeck A, de Grandmont P, Rompré PH, Lavigne GJ. Effect of an adjustable mandibular advancement appliance on sleep bruxism: a crossover sleep laboratory study. *Int. J. Prosthodont.* **2009**, 22(3), 251-259.
55. Lee SJ, McCall WD Jr, Kim YK, Chung SC, Chung JW. Effect of botulinum toxin injection on nocturnal bruxism: a randomized controlled trial. *Am. J. Phys Med.Rehabil.* **2010**, 89(1), 16-23.
56. Madani AS, Abdollahian E, Khiavi HA, et al. The efficacy of gabapentin versus stabilization splint in management of sleep bruxism. *J. Prosthodont.* **2013**, 22(2), 126-131.
57. Mainieri VC, Saueressig AC, Fagondes SC, Teixeira ER, Rehm DD, Grossi ML. Analysis of the effects of a mandibular advancement device on sleep bruxism using polysomnography, the BiteStrip, the sleep assessment questionnaire, and occlusal force. *Int. J. Prosthodont.* **2014**, 27(2), 119-126.
58. Matsumoto H, Tsukiyama Y, Kuwatsuru R, Koyano K. The effect of intermittent use of occlusal splint devices on sleep bruxism: a 4-week observation with a portable electromyographic recording device. *J. Oral Rehabil.* **2015**, 42(4), 251-258.
59. Nixdorf DR, Heo G, Major PW. Randomized controlled trial of botulinum toxin A for chronic myogenous orofacial pain. *Pain*. **2002**, 99(3), 465-473.
60. Ohmure H, Kanematsu-Hashimoto K, Nagayama K, et al. Evaluation of a Proton Pump Inhibitor for Sleep Bruxism: A Randomized Clinical Trial. *J. Dent. Res.* **2016**, 95(13), 1479-1486.
61. Ondo WG, Simmons JH, Shahid MH, Hashem V, Hunter C, Jankovic J. Onabotulinum toxin-A injections for sleep bruxism: A double-blind, placebo-controlled study. *Neurology*. **2018**, 90(7), e559-e564.
62. Patel AA, Lerner MZ, Blitzer A. IncobotulinumtoxinA Injection for Temporomandibular Joint Disorder. *Ann. Otol. Rhinol. Laryngol.* **2017**, 126(4), 328-333.
63. Redaelli A. Botulinum Toxin A in bruxers. One year experience. *Saudi Med. J.* **2011**, 32(2), 156-158.
64. Saletu A, Parapatics S, Anderer P, Matejka M, Saletu B. Controlled clinical, polysomnographic and psychometric studies on differences between sleep bruxers and controls and acute effects of clonazepam as compared with placebo. *Eur. Arch. Psychiatry Clin. Neurosci.* **2010**, 260(2), 163–174.
65. Sato M, Iizuka T, Watanabe A, et al. Electromyogram biofeedback training for daytime clenching and its effect on sleep bruxism. *J. Oral Rehabil.* **2015**, 42(2), 83-89.
66. Shim YJ, Lee MK, Kato T, Park HU, Heo K, Kim ST. Effects of botulinum toxin on jaw motor events during sleep in sleep bruxism patients: a polysomnographic evaluation. *J. Clin. Sleep Med.* **2014**, 10(3), 291-298.
67. Shim YJ, Lee HJ, Park KJ, Kim HT, Hong IH, Kim ST. Botulinum Toxin Therapy for Managing Sleep Bruxism: A Randomized and Placebo-Controlled Trial. *Toxins*. **2020**, 12(3), 168-178.
68. Singh PK, Alvi HA, Singh BP, et al. Evaluation of various treatment modalities in sleep bruxism. *J. Prosthet. Dent.* **2015**, 114(3), 426-431.
69. Lee SJ, McCall WD Jr, Kim YK, Chung SC, Chung JW. Effect of botulinum toxin injection on nocturnal bruxism: a randomized controlled trial. *Am. J. Phys. Med. Rehabil.* **2010**, 89(1), 16-23.
70. da Silva Ramalho JA, Palma LF, Ramalho KM, Tedesco TK, Morimoto S. Effect of botulinum toxin A on pain, bite force, and satisfaction of patients with bruxism: A randomized single-blind clinical trial comparing two protocols. *Saudi Dent. J.* **2023**, 35(1), 53-60.
71. Sumiya M, Mizumori T, Kobayashi Y, Inano S, Yatani H. Suppression of sleep bruxism: effect of electrical stimulation of the masseter muscle triggered by heart rate elevation. *Int. J. Prosthodont.* **2014**, 27(1), 80-86.
72. Takahashi H, Masaki C, Makino M, et al. Management of sleep-time masticatory muscle activity using stabilisation splints affects psychological stress. *J. Oral Rehabil.* **2013**, 40(12), 892-899.
73. Valiente López M, van Selms MK, van der Zaag J, Hamburger HL, Lobbezoo F. Do sleep hygiene measures and progressive muscle relaxation influence sleep bruxism? Report of a randomised controlled trial. *J. Oral Rehabil.* **2015**, 42(4), 259-265.
74. von Lindern JJ, Niederhagen B, Bergé S, Appel T. Type A botulinum toxin in the treatment of chronic facial pain associated with masticatory hyperactivity. *J. Oral Maxillofac. Surg.* **2003**, 61(7), 774-778.
75. Yurttutan ME, Tütüncüler Sancak K, Tüzüner AM. Which Treatment Is Effective for Bruxism: Occlusal Splints or Botulinum Toxin?. *J. Oral Maxillofac. Surg.* **2019**, 77(12), 2431-2438.
76. Zhang LD, Liu Q, Zou DR, Yu LF. Occlusal force characteristics of masseteric muscles after intramuscular injection of botulinum toxin A(BTX - A)for treatment of temporomandibular disorder. *Br. J. Oral Maxillofac. Surg.* **2016**, 54(7), 736-740.
77. Gates, M.; Gates, A.; Pieper, D.; Fernandes, R.M.; Tricco, A.C.; Moher, D.; Brennan, S.E.; Li, T.; Pollock, M.; Lunny, C. Reporting Guideline for Overviews of Reviews of Healthcare Interventions: Development of the PRIOR Statement. *Br. Med. J.* **2022**, *378*, e070849.
